# Purification and Characterization of Plantaricin LPL-1, a Novel Class IIa Bacteriocin Produced by *Lactobacillus plantarum* LPL-1 Isolated From Fermented Fish

**DOI:** 10.3389/fmicb.2018.02276

**Published:** 2018-09-28

**Authors:** Yao Wang, Yuxuan Qin, Qing Xie, Ying Zhang, Jinrong Hu, Pinglan Li

**Affiliations:** Beijing Advanced Innovation Center for Food Nutrition and Human Health, College of Food Science and Nutritional Engineering, Key Laboratory of Functional Dairy, China Agricultural University, Beijing, China

**Keywords:** plantaricin LPL-1, *Lactobacillus plantarum*, characteristics, antibacterial activity, mode of action

## Abstract

Bacteriocins are ribosomally synthesized peptides or proteins possessing antibacterial activity against foodborne pathogens and spoilage bacteria. A novel bacteriocin, plantaricin LPL-1 was determined as a class IIa bacteriocin according to the YGNGV motif, and producer strain *Lactobacillus plantarum* LPL-1 was identified based on physio-biochemical characteristics and 16S rDNA sequence. The novel bacteriocin, plantaricin LPL-1 was purified by salt precipitation, cation exchange, gel filtration, and reverse phase high-performance liquid chromatography (RP-HPLC). The molecular mass of plantaricin LPL-1 was 4347.8467 Da by Matrix-assisted laser desorption/ionization time-of-flight mass spectrometry (MALDI-TOF-MS) analysis and entire amino acid sequence of plantaricin LPL-1 was VIADKYYGNGVSCGKHTCTVDWGEAFSCSVSHLANFGHGKC. Plantaricin LPL-1 possessed the merits of easy degradation by proteases, wide pH stability (2–10), high thermal stability (121°C, 20 min), surfactants stability and bactericidal activity against foodborne spoilage and pathogens bacteria. The mode action and membrane permeabilization of plantaricin was identified. The information of plantaricin LPL-1 indicated that it is not only a novel class IIa bacteriocin, but also a promising natural and safe biologic preservative for the food preservation industry.

## Introduction

Bacteriocins are ribosomally synthesized peptides or protein with antibacterial activity toward strains either within the same species or across different genera (De Vuyst and Leroy, 2007), although, an increasing number of novel bacteriocins against foodborne pathogens have been reported (Liu et al., 2015; Yi et al., 2016; Zhao et al., 2016). Recently, the bacteriocins produced by lactic acid bacteria (LAB) have gained great attention because of their potential as safe and effective bio-preservative agents. It is also well-known that the bacteriocins from LAB are not only harmless to the human body, but also capable of inhibiting the growth of foodborne pathogens, such as *Listeria monocytogenes*, *Escherichia coli*, and *Staphylococcus aureus* (Liu et al., 2016, 2017; Yi et al., 2016), so that bacteriocins have been widely applied in many countries for natural food preservation and safety (Cotter et al., 2013; Yang et al., 2014). For example, the bacteriocin nisin, which was found in 1933 in New Zealand, has been allowed to be used in 48 countries since its first marketed in England in 1953 (Tagg et al., 1995).

Based on their primary structure, molecular mass, thermal stability, mode of action, and genetic properties, the bacteriocins produced by LAB have been divided into four classes (Klaenhammer, 1993): class I (<5 kDa), heat-stable and lanthionine-containing bacteriocins; class II (<10 kDa), heat-stable and non-lanthionine-containing bacteriocins; class III (>30 kDa), heat sensitive, protein-like bacteriocins; and class IV, complex bacteriocins containing lipid or carbohydrate moieties. Among these classes, class II has been divided into four subclasses. Class IIa bacteriocins have a conserved YGNGV motif and a disulfide bond linkage in the N-terminal region that is essential for strong inhibitory effect on *L. monocytogenes* as well as other food spoilage and pathogenic bacteria (Perez et al., 2014). Moreover, these class IIa bacteriocins could be degraded by gastrointestinal proteases (Abbasiliasi et al., 2017). Therefore, class IIa bacteriocins are promising candidates as bio-preservatives, and are considered as a good candidate to replace chemical preservatives (Gao et al., 2010).

It is well known that bacteriocin producers exist widely in fermented food, and LAB are considered as the dominant microorganism in fermented fish products (Paludan-Muller et al., 2002a; Liu et al., 2011). A variety of bacteriocin producers isolated from fermented fish have been reported, including *Enterococcus faecium* CN-25 (Sonsa-Ard et al., 2015), *Pediococcus pentosaceus* CFF4 (Peng et al., 2017), *E. faecium* NKR-5-3 (Ishibashi et al., 2012), *Lactobacillus plantarum* PMU 33 (Paludan-Muller et al., 2002b). Although previous studies have identified the bacteriocin and producer cells as valuable to the food industry, the reports concerning practical application in the food industry are relatively rare (Hata et al., 2010). Therefore, more novel bacteriocins that have potential use as natural and safe food preservatives in the food industry remain to be explored.

In the present paper, a novel class IIa bacteriocin, plantaricin LPL-1 produced by *L. plantarum* LPL-1 isolated from fermented fish in Beijing, China, was purified and characterized. Furthermore, we describe the production, physicochemical characterization, and mode of action of plantaricin LPL-1. This study provides insight into the potential use of bacteriocins as a food preservative in the food industry.

## Materials and Methods

### Samples, Bacterial and Growth Conditions

Samples of fermented fish products prepared from sturgeon fish was collected from Beijing innovation team of sturgeon and trout in Beijing, People’s Republic of China. Lactic acid bacteria were cultured in MRS broth (AoBoxing, Beijing, China) at 37°C. The indicator strain, *L. monocytogenes* 54002 stored in lab, was cultured in TSYEB broth at 37°C. The medium used for culture of other bacteria was showed in **Table 1**. All bacteria were stored at −80°C in proper culture medium with 20% (v/v) glycerol.

**Table 1:** Antibacterial spectrum of plantaricin LPL-1.

Indicator strain	Source	Media	Activity (mm)	MIC (μg/mL)
**Gram-positive bacteria**				
*Listeria monocytogenes* 54002	NICPBP	TSYEB	16.4 ± 0.23	16
*L. monocytogenes* 19113(3a)	ATCC	TSYEB	16.32 ± 0.43	16
*L. monocytogenes* 19114(4a)	ATCC	TSYEB	16.21 ± 0.35	16
*Staphylococcus aureus* 13565	ATCC	TSB	9.12 ± 0.42	32
*S. aureus* 6538	CGNCC	TSB	8.21 ± 0.21	32
*S. aureus* 26112	CVCC	TSB	8.31 ± 0.42	32
*Enterococcus faecalis* M2	Lab	MRS	6.32 ± 0.52	32
*E. faecalis*	Lab	MRS	7.98 ± 0.11	32
*Lactobacillus delbrueckii subsp.lactis*	Lab	MRS	18.81 ± 0.12	16
*Lactobacillus plantarum* S-35	Lab	MRS	17.32 ± 0.33	16
*L. plantarum* γ-35	Lab	MRS	16.98 ± 0.21	16
*Lactobacillus bulgaricus*	Lab	MRS	14.32 ± 0.29	16
*Lactobacillus salivarius*	Lab	MRS	15.11 ± 0.32	16
*Lactococcus lactis* NZ9000	Lab	MRS	14.43 ± 0.21	16
*L. lactis* MG1363	Lab	MRS	14.12 ± 0.39	16
*Bacillus amyloliquefaciens*	Lab	LB	7.43 ± 0.32	32
*B. pumilus*	Lab	LB	7.67 ± 0.21	32
**Gram-negative bacteria**				
*Escherichia coli* DH5α	Lab	LB	0	
*E. coli* BL21	Lab	LB	0	
*E. coli* BW25113	Lab	LB	0	
*E. coli* JM109	Lab	LB	0	
**Fungi**				
*Saccharomyces cerevisiae*	Lab	YPD	0	
*Pichia pastoris* GS115	Lab	YPD	0	

*TSYEB media (Tryptone 17 g/L, Soya Peptone 3 g/L, Yeast Extract 6 g/L, Sodium Chloride 5 g/L, Dipotassium Phosphate 2.5 g/L, Glucose 2.5 g/L, and pH 7.3 ± 0.2); TSB media (Tryptone 17 g/L, Soytone 3 g/L, Glucose 2.5 g/L, Sodium Chloride 5 g/L, Dipotassium Phosphate 2.5 g/L, and pH 7.3 ± 0.2); MRS media (Protease peptone 10 g/L, Beef extract 10 g/L, Yeast extract 5 g/L, Glucose 20 g/L, Tween 80 1 g/L, Dipotassium Phosphate 2 g/L, Sodium acetate 5g/L, Ammonium citrate 2 g/L, Magnesium Sulfate 0.2 g/L, Manganese Sulfate 0.05 g/L, and pH 6.5 ± 0.2); LB media (Tryptone 10 g/L, Yeast extract 5 g/L, NaCl 10 g/L, and pH 7.3 ± 0.2); and YPD media (Yeast Extract 10.0 g/L, Peptone 20 g/L, Glucose 20 g/L, and pH 6.5 ± 0.2).*

### Screening for Bacteriocin-Producing LAB Strains

Lactic acid bacteria strains from samples were spread onto the surface of MRS-agar plates, and the plates were incubated at 37°C for 24–48 h. A total of 616 single colonies were inoculated anaerobically into 5 mL of MRS broth for 24 h at 37°C. The supernatant was obtained by centrifugation of culture at 8000 × *g* for 20 min at 4°C and readjusted to pH 6.8 ± 0.2 using 1 M NaOH. The cell-free supernatants (CFS) of all isolated LAB strains filtered through sterile 0.22 μm filters were used for detection of antibacterial activities by agar well diffusion assay. The *L. monocytogenes* 54002 was used as the indicator strain. The antibacterial activities of CFS against indicator strain were determined by measuring the diameters of inhibition zones with vernier caliper. The selected strains (31 strains) with relatively higher antibacterial activity were further tested using other food spoilage bacteria (*L. monocytogenes*, *S. aureus*, *Bacillus amyloliquefaciens*, and *Bacillus pumilus*) as indicator strains. After incubation for 12 h at 37°C, the diameter of inhibition zones was measured, and a strain with relatively larger inhibition zone was selected for subsequent studies.

### Identification of the Bacteriocin-Producing Strain

In addition to the catalase reaction and carbohydrate fermentation patterns, the bacteriocin-producing strain was characterized and identified based on morphological, biochemical, and physiological characteristics (Vos et al., 2009). Morphological and physiological identification was examined by Gram staining, shape, and motility through light microscopy. Biochemical identification was based upon the ability to grow at different temperatures and salt concentrations. Finally, genotype identification was confirmed according to 16S rRNA sequence analysis. The PCR reaction primers were as follows: 16S rRNA–F: 5′–AGAGTTTGATCMTGGCTCAG–3′ and 16 rRNA–R: 5′–TACGGYTACCTTGTTACGACTT–3′. Briefly, the whole genome was extracted using a genome extraction kit (TianGen, Beijing, China) and was amplified with initial denaturation for 10 min at 95°C, followed by 30 cycles of 30 s at 94°C, 30 s at 50°C, and 1 min at 72°C, with final extension for 10 min at 72°C. The PCR products were purified by PCR purification kit (TianGen, Beijing, China) and were sequenced by Sangon Biotech (Shanghai, China). The results of DNA sequencing were blasted against the GenBank database^1^. Subsequently, software MEGA 7.0 was used for phylogenetic analysis.

### Dynamics of Growth and Antibacterial Activity of Bacteriocin

*Lactobacillus plantarum* LPL-1 was cultured in 50 mL MRS broth until OD_600_ = 0.4, then inoculated into 1 L MRS broth (pH 6.5) at 0.5% level (v/v) inoculum for 64 h at 37°C without agitation. During this process, culture samples were taken every 4 h to determine the cell density measured at 600 nm, pH of the culture and antibacterial activity against an indicator strain (*L. monocytogenes* 54002). The antibacterial activity of crude plantaricin LPL-1 was assessed by agar-well diffusion method (Mayr-Harting et al., 1972). The activity was expressed as arbitrary units per milliliter of culture medium (AU/mL) and one AU was defined as the reciprocal of the highest two-fold dilution exhibiting a clear inhibition zone of the indicator strain (Deraz et al., 2005).

### Purification Process of Plantaricin LPL-1

*Lactobacillus plantarum* LPL-1 was statically cultured in 50 mL MRS broth to OD_600_ = 0.4, at which point 0.5% (v/v) of the above culture was inoculated into 1000 mL MRS broth and then incubated for 32 h at 37°C without agitation. The fermentation culture was centrifuged at 8000 × *g* for 20 min at 4°C to remove bacterial cells. Then, ammonium sulfate was added to the collected supernatant to 70% saturation with stirring at 4°C. The mixture was centrifuged at 8000 × *g* for 20 min at 4°C to obtain supernatant containing crude bacteriocin. The crude bacteriocin was suspended in 20 mL distilled water and the activity against the indicator strain was assayed by agar-well diffusion method (Mayr-Harting et al., 1972). The sample was stored in 30% (v/v) glycerol at −20°C for further analyses.

The SP-Sepharose Fast Flow cation exchange column (1.6 × 2.0 cm, GE, Uppsala, Sweden) incorporated with ÄKTA^TM^ chromatography system (GE, Sweden and United States) was equilibrated with 20 mM phosphate buffer (pH 5.5). After equilibration, the crude bacteriocin was filtered through a 0.22 μm filter membrane (Merck Millipore, United States) and was loaded onto column. The loaded column was washed with 20 mM phosphate buffer (pH 5.5) and active fractions were eluted with a linear gradient from 0 to 1 M NaCl for 90 min. The flow rate was 1 mL/min and absorbance was 280 nm. The collected absorption peaks were evaluated for antibacterial activity and the active fraction was stored for further purification and analyses.

Similarly, the active fraction, which was collected by cation exchange chromatography and filtered through 0.22 μm filter membrane, was loaded onto a Sephadex G25 column (2.6 × 10.0 cm, GE, Uppsala, Sweden) equilibrated with 20 mM phosphate buffer (pH 5.5). The column was eluted with the same buffer and the different absorption peaks were collected. The flow rate was 0.5 mL/min and the absorbance was 280 nm. The collected fractions were evaluated for antibacterial activity and the active fraction was dialyzed in distilled water for further purification.

The active fraction purified from gel filtration chromatography process was loaded onto a C18 reverse-phase column (5 μm, 4.6 × 250 mm, Agilent, CA, United States) and incorporated in reverse phase high-performance liquid chromatography (RP-HPLC) system (Agilent, CA, Untied States) by a liner gradient elution with 95% water-acetonitrile (5–95%) containing 0.1% trifluoroacetic acid (TFA) in 30 min. The flow rate was 0.5 mL/min and the absorbance was monitored at 280 nm. The purified active fraction was evaluated for antibacterial activity and identified molecular mass.

The concentration of protein in every process of purification was determined by bicinchoninic acid (BCA) kit (Thermo Fisher Scientific, MA, United States) as described by instructions.

### Determination of Molecular Mass

The molecular mass of plantaricin LPL-1 was determined by ABI 4700 matrix-assisted laser desorption/ionization time-of-flight (MALDI-TOF) mass spectrometry (Applied Biosystems, Foster city, United States). The sample was spotted on a target plate, left to dry, and α-cyano-4-hydroxycinnamic acid (Sigma, United States) matrix solution was spotted on the same target plate. Spectrum was then operated in positive ion mode for MALDI analysis.

### Antibacterial Spectrum and Inhibitory Concentration of Plantaricin LPL-1

The partially purified plantaricin LPL-1 preparation from cation exchange chromatography was used to determine the antibacterial spectrum against indicator organisms including food spoilage bacteria, foodborne pathogens, and fungi (**Table 1**). The diameter of inhibition zones was measured by Vernier caliper. The minimum inhibitory concentration (MIC) of plantaricin LPL-1 against indicator strains was specified by the serial two-fold method. Briefly, the stock solution of 512 μg/mL plantaricin LPL-1 was 2-fold serially diluted with 20 mM phosphate buffer (pH 6.5) and 100 μL of each dilution was added into the wells. The cultured different indicator strains were spread on the plate and the plate was incubated at 37°C for 16 h to determine MIC values. The MIC was defined as the lowest concentration of plantaricin LPL-1 that exhibited a clear inhibition zone of indicator strains.

### Effects of Enzyme, pH, Temperature and Surfactant on Activity of Plantaricin LPL-1

The sensitivity of purified plantaricin LPL-1 toward various proteases were individually evaluated by incubating 360 μL of bacteriocin (256 μg/mL) with 40 μL of the following enzymes (Sigma) at a final concentration of 1 mg/mL: pepsin (pH 3.0), papain (pH 6.5), proteinase K (pH 7.5), trypsin (pH 7.6) and chymotrypsin (pH 7.8). After 3 h of incubation at appropriate the temperature (**Table 3**), the activity against indicator strain (*L. monocytogenes* 54002) was determined. Non-enzymic treatment was used as control test.

The pH stability of purified plantaricin LPL-1 (256 μg/mL) was determined by adjusting pH with HCl and NaOH in a range from 2.0 to 10.0. After 3 h of incubation at 37°C, the pH was readjusted to 6.0 and the activity of plantaricin LPL-1 was assayed.

To determine thermal stability of plantaricin LPL-1 (256 μg/mL), the samples were exposed in a thermostatic water bath at temperatures of 60, 80 and 100°C for 20 and 30 min, and in an autoclave at 121°C for 20 min. The residual activity against indicator was measured and samples at 25°C were used as control.

The effect of surfactants on plantaricin LPL-1 (256 μg/mL) was examined by adding 1% (v/v) concentration of EDTA, Tween 20, Tween 80, and urea, separately. After 3 h of incubation at 37°C, the remaining activity against indicator was determined and untreated samples were used as a control test.

### Mode of Action

To determine the mode of action of plantaricin LPL-1, the indicator strain, *L. monocytogenes* 54002 was cultured in TSYEB broth for 16 h at 37°C. Cells were harvested and resuspended in sterile 20 mM sodium phosphate buffer to give 10^8^ CFU/mL approximately. Bacteriocin was added for final concentration of 256 μg/mL, while a sample without added bacteriocin was used as control. All samples were incubated for 5 h at 37°C. The optical density (600 nm) and viable cells were monitored every 1 h (Deraz et al., 2007).

### Membrane Permeabilization

The fluorescence was detected using RT-PCR instrument combined with 5 μM SYTOX^TM^ Green Stain (Invitrogen, United States). Cells were resuspended in sterile 20 mM sodium phosphate buffer to give 10^6^ CFU/mL approximately. Bacteriocin was added at final concentration of 256 and 128 μg/mL, respectively, while a sample with added sodium phosphate buffer was used as control. Meanwhile, the treated cells were examined using confocal laser scanning microscopy (CLSM; Leica, United States) combined with 5 μM SYTOX^TM^ Green Dead Cell Stain.

### Statistical Analysis

All experiments were performed in triplicate. The data were tested by ANOVA and Duncan’s test with SPSS 23.0. All results were presented as mean ± standard deviations (SD) and *p*-value < 0.05 was considered statistically significant.

## Results

### Screening of Bacteriocin-Producing LAB Strains

A total of 616 potential bacteriocin-producing LAB strains isolated from fermented fish products were incubated in MRS broth. The CFS of 31 strains showed antibacterial activity against indicator strain in primary screening process (**Supplementary Table S1**). After secondary screening process, the CFS of three strains exhibited higher antibacterial activity against other food spoilage bacteria. Among three strains, strain LPL-1 possessed the highest antibacterial activity was selected as target strain.

### Identification of Strain LPL-1

**FIGURE 1:**
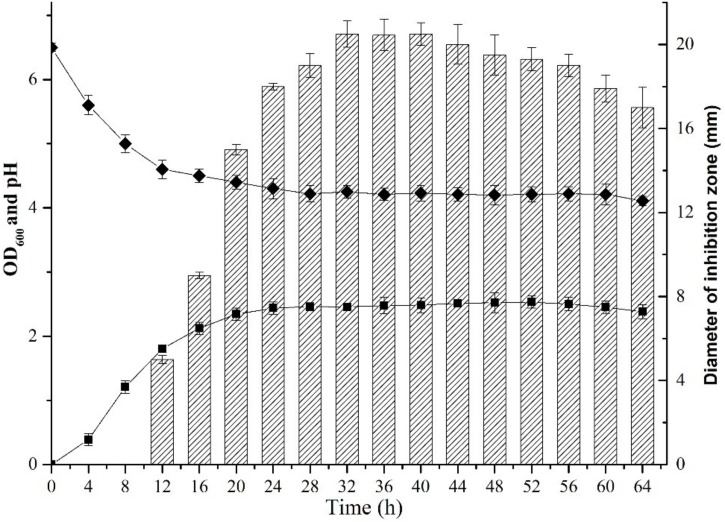
Dynamics of growth and production of plantaricin LPL-1 from *L. plantarum* LPL-1. Growth curve (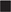); pH (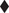); and Activity against indicator (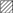).

Strain LPL-1, isolated from fermented fish, was characterized as a Gram-positive, rod-shaped, catalase-negative, and facultative anaerobic strain without spore formation. It could grow at 15°C but not at 45°C and in 3.0% NaCl but not in 6.0% NaCl. In addition, it could produce acid from glucose, and was capable of metabolizing fructose, galactose, glucose, lactose, maltose, sucrose, mannose, and xylose. Furthermore, the complete genome and the 1488 bp 16S rRNA nucleotide sequence was identified (GenBank accession number CP021997.1). A neighbor-joining phylogenetic tree (**Supplementary Figure S1**) was constructed by sequence alignment using MEGA 7.0 (Kumar et al., 2016) (**Supplementary Table S2**), which showed extremely high similarity (identity ≥ 99%, E value = 0) to *L. plantarum* (**Figure 1**).

### Dynamics of Growth and Bacteriocin Production

Dynamics of growth, bacteriocin production, and pH of *L. plantarum* LPL-1 were examined in MRS broth at 37°C (**Figure 1**). During growth, LPL-1 entered exponential growth phase at 4 h, remained in stationary phase from 20 to 52 h and reached decline phase after 52 h. The activity of bacteriocin against the indicator strain (*L. monocytogenes* 54002) was determined by the size of the diameter of inhibition zone. Accordingly, the bacteriocin was produced at 12 h during the late exponential growth phase and reached maximum at 32 h during stationary phase. The pH of the medium was decreased from 6.5 to 4.02.

### Purification of Plantaricin LPL-1

The plantaricin LPL-1 was obtained by ammonium sulfate precipitation, cation exchange column, gel-filtration chromatography, and RP-HPLC. The results of different purification steps were shown in **Table 2**.

**Table 2:** Purification of plantaricin LPL-1.

Purification Stage	Volume (mL)	Total protein (mg)	Total activity (AU)	Specific activity (AU/mg)	Purification fold	Recovery (%)
Culture supernatant	1000	4,002	256,000	63.96	1	100
Ammonium sulfate precipitation	20	1,245	204,000	163.85	2.56	79.68
SP-Sepharose Fast Flow	10	38.7	92,000	2,377.26	37.16	35.93
Sephadex G10	4	5.4	21,300	3,944.44	61.66	8.32
RP-HPLC	0.3	0.96	5,320	5,541.66	86.63	2.08

**FIGURE 2:**
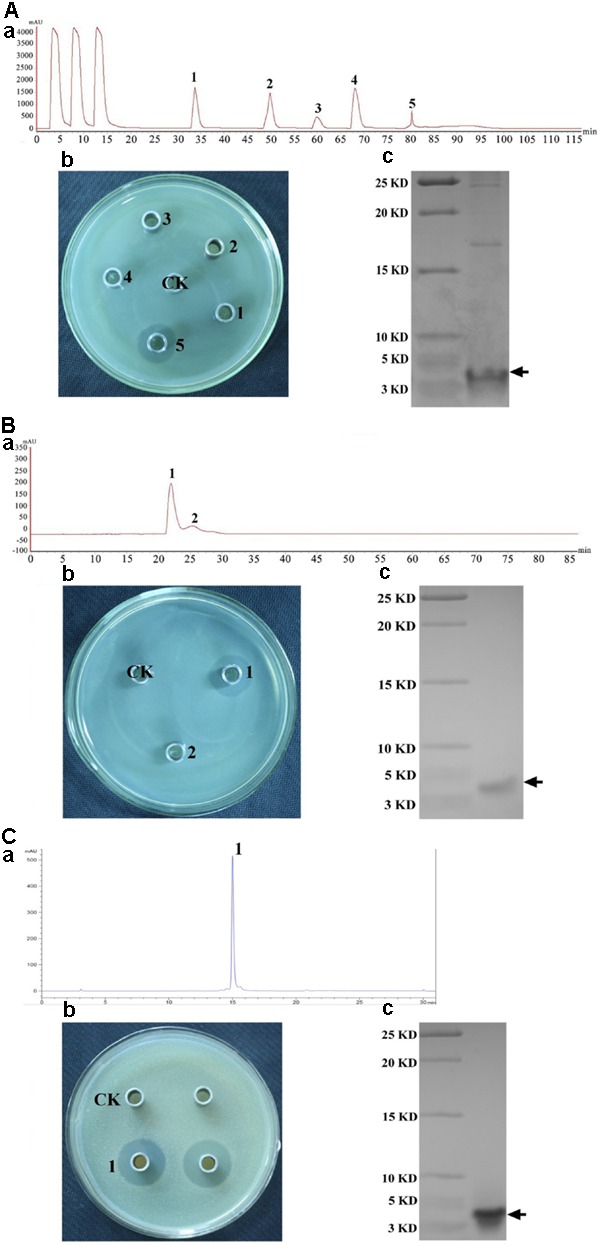
Purification of plantaricin LPL-1 produced by *L. plantarum* LPL-1 by chromatography column. **(A)** Cation exchange column; **(B)** gel filtration chromatography; and **(C)**: RP-HPLC process. **(a)** Process of purification; **(b)**: antibacterial activity of absorbance peaks against indicator strain compared with CK (control test) by agar well diffusion assay; and **(c)**: Tricine-SDS-PAGE of purified activity fraction.

Crude plantaricin LPL-1 was concentrated from culture supernatant by ammonium sulfate precipitation. The purification fold was 2.56 and recovery was 79.68%. The crude bacteriocin was purified with an SP-Sepharose Fast Flow cation exchange column (**Figure 2Aa**). Peak 5 showed antibacterial activity by agar well diffusion method (**Figure 2Ab**) and a dominant band was about 4.0 kDa by Tricine-SDS-PAGE (**Figure 2Ac**). At this purification step, approximately 37.16-fold purification and 35.96% recovery were achieved. The active fraction was subjected to Sephadex G10 gel-filtration chromatography (**Figure 2Ba**). Peak 1 was also capable of inhibiting growth of indicator strain (**Figure 2Bb**) and the dominant band was further purified (**Figure 2Bc**). The antibacterial activity increased 61.66-fold, and 8.32% of the initial activity was recovered. Following the final purification by RP-HPLC, the process showed a single peak (**Figure 2Ca**) and the peak was active against indicator strain (**Figure 2Cb**), and the plantaricin LPL-1 was further purified (**Figure 2Cc**). This purification step increased 86.63-fold activity and the recovery was 2.08%.

### Molecular Mass and Sequence of Plantaricin LPL-1

**FIGURE 3:**
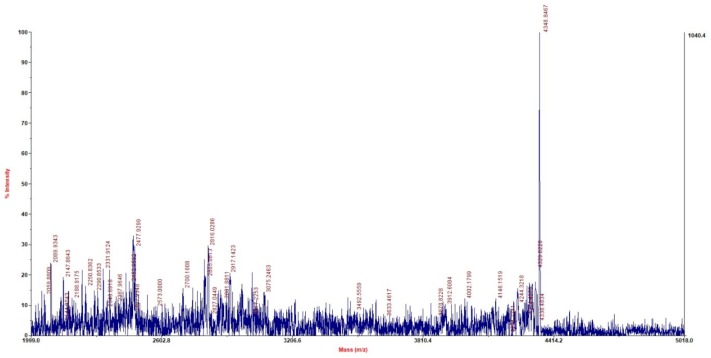
Mass spectrum of plantaricin LPL-1 by MALDI-TOF-MS.

**FIGURE 4:**
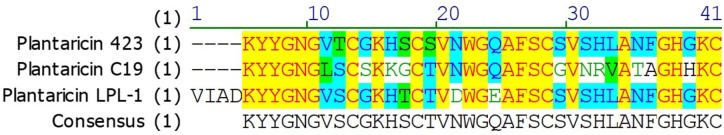
Alignment of reported class IIa bacteriocin, plantaricin 423, plantaricin C19, and plantaricin LPL-1 from *L. plantarum*. Alignments were obtained using Vector NTI 11.5 with default settings.

MALDI-TOF-MS analysis of purified plantaricin LPL-1 from RP-HPLC procedure showed that its molecular mass was 4347.8467 Da (**Figure 3**). Furthermore, the complete sequence of prebacteriocin was identified (GenBank accession number CP021998) (Wang et al., 2018). However, the cleavage site between leader peptide and mature peptide was uncertain. According to the information of genome sequence and molecular mass, the entire amino acid sequence was VIADKYYGNGVSCGKHTCTVDWGEAFSCSVSHLANFGHGKC. The observed molecular (4347.8467 Da) mass was about 2 Da smaller than the calculated (4349.84 Da) because of the formation of a disulfide bond. This bond is essential for the activity against the indicator strain (Drider et al., 2006). According to the N-terminal conserved YGNGV motif of mature class IIa bacteriocin, plantaricin LPL-1 belongs to class IIa bacteriocin. Hence, this study is the first to report the novel amino acid sequence and molecular mass of plantaricin to be 4347.8467 Da. The sequence of plantaricin LPL-1 did not show homology with the known bacteriocins by protein BLAST against the GenBank (see footnote 1) and the Antimicrobial Peptide Database^2^. Aligned with reported class IIa bacteriocin, plantaricin 423 (van Reenen et al., 2010), plantaricin C19 (Atrih et al., 2001) from *L. plantarum*, plantaricin LPL-1 possessed novel amino acid sequence (**Figure 4**). Therefore, plantaricin LPL-1 is a novel class IIa bacteriocin from *L. plantarum*.

### Antibacterial Spectrum of Plantaricin LPL-1

The antibacterial spectrum of plantaricin LPL-1 is shown in **Table 1**. Plantaricin LPL-1 exhibited significant antibacterial activity against Gram-positive bacteria, such as *S. aureus*, *L. monocytogenes*, *B. pumilus*, *B. amyloliquefaciens*, *E. faecalis*, *L. plantarum*, *Lactobacillus delbrueckii*, *Lb. bulgaricus*, *Lactobacillus Salivarius*, and *L. lactis*. However, the purified plantaricin LPL-1 did not show antibacterial activity against *E. coli*, *Pichia pastoris*, or *Saccharomyces cerevisiae*.

### Characteristics of Plantaricin LPL-1

After treatment with various enzymes, including pepsin, papain, proteinase K, trypsin, and chymotrypsin, the inhibitory activity of plantaricin LPL-1 was lost completely (**Table 3**). These enzymes exist in the human body.

**Table 3:** Effect of enzymes on antibacterial activity.

Enzyme	pH	Diameter (mm)	Residual activity (%)	Temperature (°C)
Pepsin	3	0	0	37
Papain	6.5	0	0	37
Proteinase K	7.5	0	0	37
Trypsin	7.6	0	0	25
Chymotrypsin	7.8	0	0	25
CK (control test)	6	16.32 ± 0.12	100	37

**FIGURE 5:**
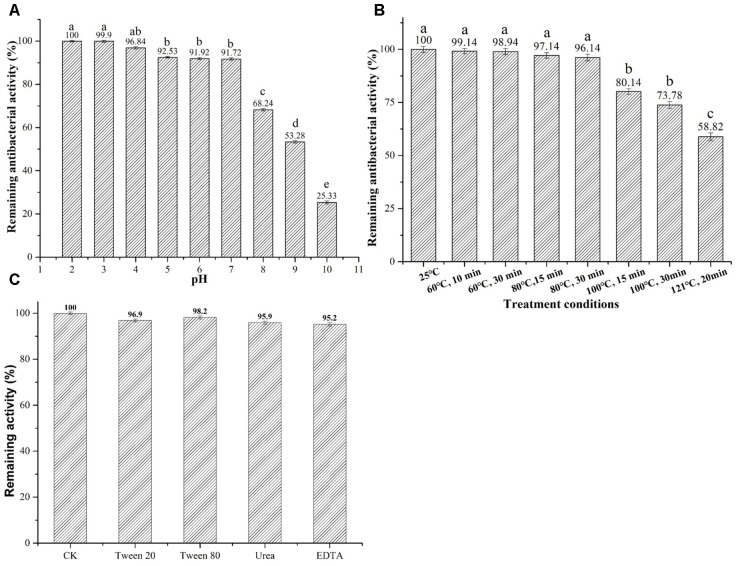
Effects of pH, temperature and surfactant on stability of plantaricin LPL-1. **(A)** pH; **(B)** temperature; and **(C)** surfactants.

Additionally, the information of pH stability revealed that plantaricin LPL-1 kept 90% of activity at pH between 2.0 and 7.0 and maintained 25% of antibacterial activity at pH 10 (**Figure 5A**).

Assessment of thermal stability demonstrated that plantaricin LPL-1 remained stable after treatment at 60, 80, and 100°C for 15 and 30 min. 58.82% inhibitory activity was observed after treatment at 120° for 20 min (**Figure 5B**).

Surfactants do not affect protein activity and play an important role in the food industry (Kralova and Sjöblom, 2009). The activity of plantaricin LPL-1 remained stable after incubation with chemical surfactants 1% (V/V) Tween 20, Tween 80, urea, and EDTA at 37°C for 3 h (**Figure 5C**).

### Mode of Action

**FIGURE 6:**
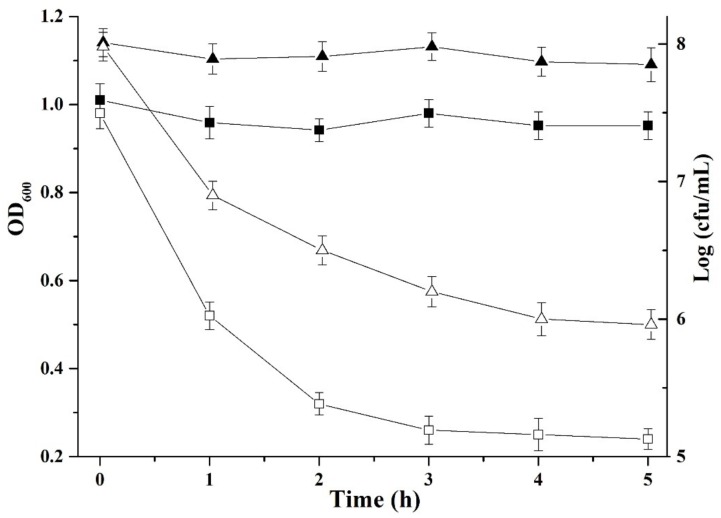
Mode of action of plantaricin LPL-1 against the indicator cells of *Listeria monocytogenes* 54002. (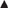): Viable cell count without bacteriocin; (Δ): viable cell count with bacteriocin; (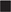): optical density at 600 nm without bacteriocin; and (□): optical density at 600 nm with bacteriocin.

To understand the mechanism of plantaricin LPL-1 against *L. monocytogenes* 54002, it is essential to analyze the mode of action. The number of viable cells (2.11 Log_10_ cfu) and optical density (0.69 units) decreased after 3 h (**Figure 6**). This indicates that plantaricin LPL-1 induced cell lysis via bactericidal activity.

### Membrane Permeabilization

**FIGURE 7:**
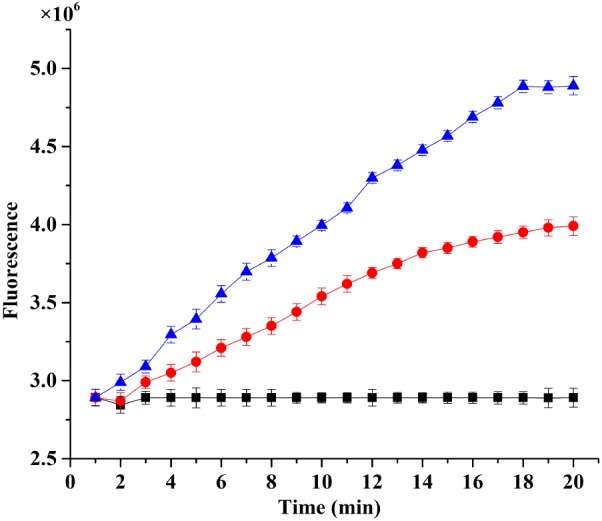
Analysis of membrane permeabilization *Listeria monocytogenes* 54002 using RT-PCR instrument combined with SYTOX^TM^ Green Dead Cell Stain. (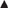): Treatment with 128 μg/mL plantaricin; (•): treatment with 256 μg/mL plantaricin; and (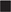): control test with sodium phosphate buffer.

**FIGURE 8:**
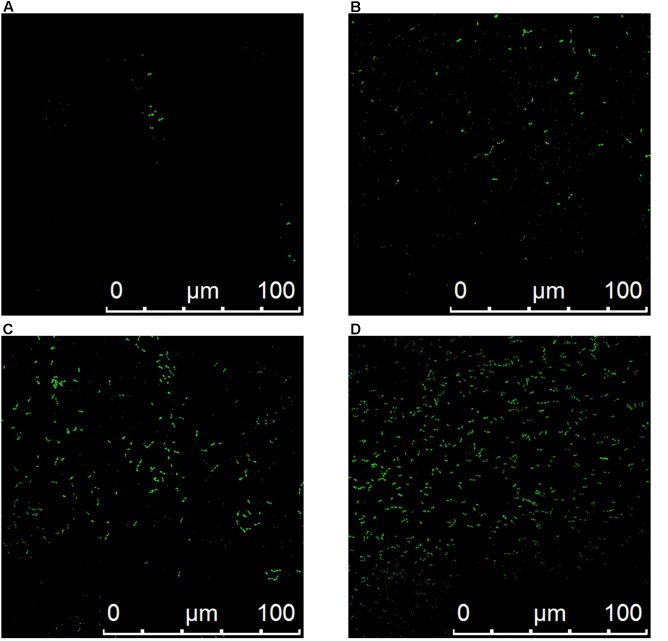
CLSM images of dead *Listeria monocytogenes* 54002. **(A)** Control test with sodium phosphate buffer and treatment with 256 μg/mL plantaricin for 10 min **(B)**, 20 min **(C)** and 30 min **(D)**.

The fluorescence intensity of treatment with plantaricin LPL-1 was increased during the membrane damage of *L. monocytogenes* 54002. However, the fluorescence intensity of control test remained stable (**Figure 7**). As showed in **Figure 8**, the amounts of dead cells were increased gradually during incubation with 256 μg/mL plantaricin LPL-1. The results suggested that the SYTOX Green stain bound nucleic acids and cell membrane was damaged.

## Discussion

In the previous study (Wang et al., 2018), the complete genome sequence of *L. plantarum* LPL-1 and biosynthetic mechanism of plantaricin LPL-1 were analyzed. The plantaricin LPL-1 was just purified by absorption and desorption of pH, and cation exchange column, but it did not prove that plantaricin LPL-1 was a novel class IIa bacteriocin. The precursor of class IIa bacteriocin contains leader peptide and mature peptide, and the N-terminal leader peptide is cleaved to form a mature antibacterial bacteriocin. The previous study predicated the amino acid sequence of precursor and cleavage site between leader peptide and mature peptide by bioinformatic analyses. However, cleavage site and molecular mass of mature bacteriocin remained to be determined precisely by Matrix-Assisted Laser Desorption/Ionization Time of Flight Mass Spectrometry (MALDI-TOF-MS). Based on the previous bioinformatic prediction, the present study confirmed precisely the amino acid sequence of mature plantaricin LPL-1 by MALDI-TOF-MS and proved that plantaricin LPL-1 was a novel class IIa bacteriocin. Moreover, the plantaricin LPL-1 was purified by ammonium sulfate precipitation, cation exchange chromatography, gel filtration chromatography and RP-HPLC, different from the previous study (Wang et al., 2018). Also, the production, physicochemical characterization, mode of action, antibacterial spectrum, membrane permeabilization, and MIC values of plantaricin LPL-1 determined.

A wide range of new plantaricins was identified from *L. plantarum*, including plantaricin C19 (3.8 kDa) (Atrih et al., 2001), plantaricin ASM1 (5045.7 Da) (Hata et al., 2010), plantaricin Y (4.2 kDa) (Chen et al., 2014), and plantaricin 163 (3.5 kDa) (Hu et al., 2013). The molecular mass of plantaricin LPL-1 was 4347.8467 Da, which is different from those of previously studied plantaricins. Many strains of *L. plantarum*, and their bacteriocins have been identified, for example, *L. plantarum* B391 (bacteriocin B391) (Fernandes et al., 2017), *L. plantarum* JLA-9 (bacteriocin JLA-9) (Zhao et al., 2016), and *L. plantarum* ZJ2008 (plantaricin ZJ2008) (Zhu et al., 2014). Similar to the previous studies of *L. plantarum*, the strain LPL-1 was determined as *L. plantarum*, which has potential value as a starter culture due to its functional properties (Mathara et al., 2008). In the present study, *L. plantarum* LPL-1 is capable of producing bacteriocin, which highlights that it is an ideal candidate for bio-preservative in food industries. To determine the amino acid sequence, the plantaricin LPL-1 was purified. According to previous studies, several classical strategies including salt precipitation, cation exchange, gel filtration, and RP-HPLC were used as methods of purification. A series of bacteriocin, bifidocin A (Liu et al., 2015), lactocin XN8-A (Yi et al., 2016), and plantaricin JLA-9 (Zhao et al., 2016) have been purified by the mentioned procedure. Also, these techniques were used for purification of plantaricin LPL-1. The parameters of purification efficiencies are shown in **Table 2**. Specifically, the purification process of RP-HPLC provided a basis for determination of molecular mass. According to the information of complete genome sequence and mass spectrum, the amino acid sequence was determined. The sequence of plantaricin LPL-1 did not show homology with the known bacteriocins by protein BLAST against the GenBank (see footnote 2) and the Antimicrobial Peptide Database (see footnote 3); therefore, plantaricin LPL-1 is a novel member of class IIa bacteriocins as some amino acids (YGNGV motif) in the sequences shown in **Figure 4** are conserved. The common consensus of N-terminal region was KYYGNGxxCxKxxCxVxWGxAFSC.

Plantaricin LPL-1 exhibited significant antibacterial activity against Gram-positive bacteria. Similar to other class IIa bacteriocins produced by *L. plantarum*, such as plantaricin 423 (van Reenen et al., 2010), plantaricin C19 (Atrih et al., 2001) and plantaricin WHE92 (Ennahar et al., 1996), the purified plantaricin LPL-1 did not show antibacterial activity against *E. coli*, *P. pastoris*, or *S. cerevisiae*. Also, Gram-negative bacteria, such as *E. coli*, Shiga toxin-producing *E. coli* (STEC), and *Salmonella* are most frequently detected in food industry. To control food spoilage induced by Gram-negative bacteria, many authors have used bacteriocins as a part of hurdle technology (Leistner, 2000). Concretely, these Gram-negative bacteria could be controlled when bacteriocins combined with mental chelators (EDTA, sodium tripolyphosphate) or physical methods such as temperature, pH, pulsed electric field and high hydrostatic pressure (HHP) (Martínez et al., 2008; Ananou et al., 2010; Zhao et al., 2013; Khan et al., 2015; Prudêncio et al., 2015). Based on previous reports, we recommend the use of plantaricin LPL-1 combined with mental chelators or physical methods to establish a series of hurdles to control Gram-negative bacteria and fungi in different food systems. Even, the producer strain could be directly used as bio-preservative for the production of plantaricin LPL-1 and organic acid in fermented and acid products. Many bacteriocins have been shown to inhibit growth of homologous species (Tagg et al., 1995). Plantaricin LPL-1 not only possessed inhibitory activity against LAB belonging to beer spoilage microorganisms (Vaughan et al., 2012), but also against other foodborne pathogenic and spoilage bacteria. Thus, as a novel preservative candidate, plantaricin LPL-1 has the potential value as a bio-preservative in the food industry.

Recombinant DNA techniques can be used to produce sufficient protein for large-scale production. Many class IIa bacteriocins have been expressed in model strains, such as bactofencin A expressed in *E. coli* (Mesa-Pereira et al., 2017), piscicolin 126 expressed in *E. coli* (Gibbs et al., 2004), pediocin PA-1 expressed in *E. coli* (Beaulieu et al., 2007), and pediocin PA-1 expressed in *P. pastoris* (Beaulieu et al., 2005). Specifically, the activity of mesentericin Y105 expressed in *L. mesenteroides* Y 105 was improved by PCR random mutagenesis (Morisset et al., 2004). Therefore, we will improve the activity and production of plantaricin LPL-1 in *E. coli*, *P. Pastoris*, or *L. lactis* by molecular techniques.

The bacteriocin was a secondary metabolite (Bibb, 2005). To meet the demand for fermentation, the results of dynamics of growth and bacteriocin production provide a basis for large-scale production of bacteriocin LPL-1 by fermentation process. Plantaricin LPL-1 was stable in all chemical treatments. After treatment with various enzymes exist in the human body, the inhibitory activity of plantaricin LPL-1 was lost completely. Further, many plantaricins, such as plantaricin JLA-9 (Zhao et al., 2016) and plantaricin K25 (Wen et al., 2016), have similar properties in enzyme treatments. The results indicate that plantaricin LPL-1 can be digested and safely used in the food industry. However, safety for the human body remains to be determined by toxicology tests. Additionally, the information of pH stability suggested that plantaricin LPL-1 possesses potential value in acidic, neutral, and alkaline food. Assessment of thermal stability demonstrated that plantaricin LPL-1 allows for it be used in pasteurized dairy products and heat-processed food. Similar to other reported plantaricins, plantaricin JLA-9 (Zhao et al., 2016) and plantaricin J23 (Rojo-Bezares et al., 2007), plantaricin LPL-1 possessed thermal stability. The stability of plantaricin LPL-1 against surfactants makes it suitable for emulsified food. Therefore, plantaricin LPL-1 is a promising natural and safe biological preservative for the food industry.

SYTOX^TM^ Green Stain could penetrate cell membranes of dead cells, and bind nuclear acid and chromosome, but it has no effect on live cells. The membrane permeabilization of indicator strains was detected using RT-PCR instrument and CLSM. Furthermore, our team is currently investigating the mechanism of action against *L. monocytogenes* 54002. It is possible that the bactericidal activity disrupts membrane integrity and induces increased permeability. The mechanism of action for pore-forming compounds can be described by five models: barrel-stave, wedge, toroidal pore, carpet, and aggregate channel (Snyder and Worobo, 2014). To determine the mechanisms of action of plantaricin LPL-1, further investigation based on atomic force microscopy (AFM), scanning tunneling microscopy (STM), and transmission electron microscopy (TEM) will be needed. Additionally, the proton motive force (PMF), the intracellular ATP levels, and the electric conductivity should be also measured to asses membrane damage and imbalance between inside and outside cell environment (Ennahar et al., 2000).

## Conclusion

The novel class IIa bacteriocin, plantaricin LPL-1 produced by *L. plantarum* LPL-1 is first reported in this study. The entire amino acid sequence of plantaricin LPL-1 was VIADKYYGNGVSCGKHTCTVDWGEAFSCSVSHLANFGHGKC. Plantaricin LPL-1, which has a molecular mass of 4347.8467 Da, possessed bactericidal activity against foodborne spoilage and pathogenic bacteria, wide pH stability, high thermal stability, and surfactants stability, and was easily degraded by proteases. The mode action and membrane permeabilization of plantaricin was identified. Therefore, plantaricin LPL-1 is a promising natural and safe biological preservative for the food industry. Future study will investigate the bactericidal mechanism of plantaricin LPL-1.

## Author Contributions

YW and PL designed the experiments. YW, YQ, QX, YZ, and JH performed the experiments. YW and YQ analyzed the results and wrote the manuscript.

## Conflict of Interest Statement

The authors declare that the research was conducted in the absence of any commercial or financial relationships that could be construed as a potential conflict of interest.

## Footnotes

1 http://www.ncbi.nlm.nih.gov/BLAST

2 http://aps.unmc.edu/AP/main.php

## Supplementary Material

FIGURE S1 Phylogenetic tree of *L. plantarum* LPL-1 according to 16S rRNA sequence.

TABLE S1 Antibacterial activity of screened strains in primary screening process.

TABLE S2 16S rRNA sequence alignment of *Lactobacillus plantarum* LPL-1 from NCBI database.

## References

[B1] Abbasiliasi S. Tan J. S. Ibrahim T. A. T. Bashokouh F. Ramakrishnan N. R. Mustafa S. (2017). Fermentation factors influencing the production of bacteriocins by lactic acid bacteria: a review. *RSC Adv.* 7 29395–29420. 10.1039/C6RA24579J 22475936

[B2] Ananou S. Gálvez A. Martínez-Bueno M. Maqueda M. Valdivia E. (2010). Synergistic effect of enterocin AS-48 in combination with outer membrane permeabilizing treatments against *Escherichia coli* O157:H7. *J. Appl. Microbiol.* 99 1364–1372. 10.1111/j.1365-2672.2005.02733.x 16313409

[B3] Atrih A. Rekhif N. Moir A. J. Lebrihi A. Lefebvre G. (2001). Mode of action, purification and amino acid sequence of plantaricin C19, an anti-listeria bacteriocin produced by *Lactobacillus plantarum* C19. *Int. J. Food Microbiol.* 68 93–104. 10.1016/S0168-1605(01)00482-2 11545225

[B4] Beaulieu L. Groleau D. Miguez C. B. Jetté J. F. Aomari H. Subirade M. (2005). Production of pediocin PA-1 in the methylotrophic yeast *Pichia pastoris* reveals unexpected inhibition of its biological activity due to the presence of collagen-like material. *Protein Expr. Purif.* 43 111–125. 10.1016/j.pep.2005.05.012 16023368

[B5] Beaulieu L. Tolkatchev D. Jette J. F. Groleau D. Subirade M. (2007). Production of active pediocin PA-1 in *Escherichia coli* using a thioredoxin gene fusion expression approach: cloning, expression, purification, and characterization. *Can. J. Microbiol.* 53 1246–1258. 10.1139/w07-089 18026219

[B6] Bibb M. J. (2005). Regulation of secondary metabolism in *Streptomyces.* *Curr. Opin. Microbiol.* 8 208–215. 10.1016/j.mib.2005.02.016 15802254

[B7] Chen Y. S. Wang Y. C. Chow Y. S. Yanagida F. Liao C. C. Chiu C. M. (2014). Purification and characterization of plantaricin Y, a novel bacteriocin produced by *Lactobacillus plantarum* 510. *Arch. Microbiol.* 196 193–199. 10.1007/s00203-014-0958-2 24493293

[B8] Cotter P. D. Ross R. P. Hill C. (2013). Bacteriocins-a viable alternative to antibiotics? *Nat. Rev. Microbiol.* 11 95–105. 10.1038/nrmicro2937 23268227

[B9] De Vuyst L. Leroy F. (2007). Bacteriocins from lactic acid bacteria: production, purification, and food applications. *J. Mol. Microbiol. Biotechnol.* 13 194–199. 10.1159/000104752 17827969

[B10] Deraz S. F. Karlsson E. N. Hedström M. Andersson M. M. Mattiasson B. (2005). Purification and characterisation of acidocin D20079, a bacteriocin produced by *Lactobacillus acidophilus* DSM 20079. *J. Biotechnol.* 117 343–354. 10.1016/j.jbiotec.2005.02.005 15925717

[B11] Deraz S. F. Karlsson E. N. Khalil A. A. Mattiasson B. (2007). Mode of action of acidocin D20079, a bacteriocin produced by the potential probiotic strain, *Lactobacillus acidophilus* DSM 20079. *J. Ind. Microbiol. Biotechnol.* 34 373–379. 10.1007/s10295-007-0206-8 17256151

[B12] Drider D. Fimland G. Hechard Y. McMullen L. M. Prevost H. (2006). The continuing story of class IIa bacteriocins. *Microbiol. Mol. Biol. Rev.* 70 564–582. 10.1128/mmbr.00016-05 16760314 PMC1489543

[B13] Ennahar S. Aoude-Werner D. Sorokine O. Van D. A. Bringel F. Hubert J. C. (1996). Production of pediocin AcH by *Lactobacillus plantarum* WHE 92 isolated from cheese. *Appl. Environ. Microbiol.* 62 4381–4387. 8953710 10.1128/aem.62.12.4381-4387.1996 PMC168265

[B14] Ennahar S. Sashihara T. Sonomoto K. Ishizaki A. (2000). Class IIa bacteriocins: biosynthesis, structure and activity. *FEMS Microbiol. Rev.* 24 85–106. 10.1111/j.1574-6976.2000.tb00534.x 10640600

[B15] Fernandes P. Loureiro D. Monteiro V. Ramos C. Nero L. A. Todorov S. D. (2017). *Lactobacillus plantarum* isolated from cheese: production and partial characterization of bacteriocin B391. *Ann. Microbiol.* 67 433–442. 10.1007/s13213-017-1275-1

[B16] Gao Y. Jia S. Gao Q. Tan Z. (2010). A novel bacteriocin with a broad inhibitory spectrum produced by *Lactobacillus sake* C2, isolated from traditional Chinese fermented cabbage. *Food Control* 21 76–81. 10.1016/j.foodcont.2009.04.003

[B17] Gibbs G. M. Davidson B. E. Hillier A. J. (2004). Novel expression system for large-scale production and purification of recombinant class IIa bacteriocins and its application to piscicolin 126. *Appl. Environ. Microbiol.* 70 3292–3297. 10.1128/AEM.70.6.3292-3297.2004 15184123 PMC427731

[B18] Hata T. Tanaka R. Ohmomo S. (2010). Isolation and characterization of plantaricin ASM1: a new bacteriocin produced by *Lactobacillus plantarum* A-1. *Int. J. Food Microbiol.* 137 94–99. 10.1016/j.ijfoodmicro.2009.10.021 19939484

[B19] Hu M. Zhao H. Zhang C. Yu J. Lu Z. (2013). Purification and characterization of plantaricin 163, a novel bacteriocin produced by *Lactobacillus plantarum* 163 isolated from traditional chinese fermented vegetables. *J. Agric. Food Chem.* 61 11676–11682. 10.1021/jf403370y 24228753

[B20] Ishibashi N. Himeno K. Fujita K. Masuda Y. Perez R. H. Zendo T. (2012). Purification and characterization of multiple bacteriocins and an inducing peptide produced by *Enterococcus faecium* NKR-5-3 from thai fermented fish. *Biosci. Biotechnol. Biochem.* 76 947–953. 10.1271/bbb.110972 22738965

[B21] Khan A. Vu K. D. Riedl B. Lacroix M. (2015). Optimization of the antimicrobial activity of nisin, Na-EDTA and pH against gram-negative and gram-positive bacteria. *LWT Food Sci. Technol.* 61 124–129. 10.1016/j.lwt.2014.11.035

[B22] Klaenhammer T. R. (1993). Genetics of bacteriocins produced by lactic acid bacteria. *FEMS Microbiol. Rev.* 12 39–85. 10.1111/j.1574-6976.1993.tb00012.x 8398217

[B23] Kralova I. Sjöblom J. (2009). Surfactants used in food industry: a review. *J. Dispers. Sci. Technol.* 30 1363–1383. 10.1080/01932690902735561

[B24] Kumar S. Stecher G. Tamura K. (2016). MEGA7: molecular evolutionary genetics analysis version 7.0 for bigger datasets. *Mol. Biol. Evol.* 33 1870–1874. 10.1093/molbev/msw054 27004904 PMC8210823

[B25] Leistner L. (2000). Basic aspects of food preservation by hurdle technology. *Int. J. Food Microbiol.* 55 181–186. 10.1016/S0168-1605(00)00161-6 10791741

[B26] Liu G. Ren G. Zhao L. Cheng L. Wang C. Sun B. (2017). Antibacterial activity and mechanism of bifidocin a against *Listeria monocytogenes*. *Food Control* 73(Part B), 854–861. 10.1016/j.foodcont.2016.09.036

[B27] Liu G. Ren L. Song Z. Wang C. Sun B. (2015). Purification and characteristics of bifidocin A, a novel bacteriocin produced by *Bifidobacterium animalis* BB04 from centenarians’ intestine. *Food Control* 50 889–895. 10.1016/j.foodcont.2014.10.049

[B28] Liu G. Song Z. Yang X. Gao Y. Wang C. Sun B. (2016). Antibacterial mechanism of bifidocin A, a novel broad-spectrum bacteriocin produced by *Bifidobacterium animalis* BB04. *Food Control* 62 309–316. 10.1016/j.foodcont.2015.10.033

[B29] Liu S.-N. Han Y. Zhou Z.-J. (2011). Lactic acid bacteria in traditional fermented Chinese foods. *Food Res. Int.* 44 643–651. 10.1016/j.foodres.2010.12.034

[B30] Martínez V. P. Sobrino L. A. Ben O. N. Abriouel H. Lucas L. R. Valdivia E. (2008). Enhanced bactericidal effect of enterocin AS-48 in combination with high-intensity pulsed-electric field treatment against *Salmonella enterica* in apple juice. *Int. J. Food Microbiol.* 26 491–496. 10.1016/j.ijfoodmicro.2008.08.014 18829125

[B31] Mathara J. M. Schillinger U. Kutima P. M. Mbugua S. K. Guigas C. Franz C. (2008). Functional properties of *Lactobacillus plantarum* strains isolated from Maasai traditional fermented milk products in Kenya. *Curr. Microbiol.* 56 315–321. 10.1007/s00284-007-9084-6 18175177

[B32] Mayr-Harting A. Hedges A. J. Berkeley R. C. W. (1972). “Chapter VII methods for studying bacteriocins,” in *Methods in Microbiology*, eds Norris J. R. Ribbons D. W. (Cambridge, MA: Academic Press), 315–422. 10.1016/S0580-9517(08)70618-4

[B33] Mesa-Pereira B. O’Connor P. M. Rea M. C. Cotter P. D. Hill C. Ross R. P. (2017). Controlled functional expression of the bacteriocins pediocin PA-1 and bactofencin A in *Escherichia coli*. *Sci. Rep.* 7:3069. 10.1038/s41598-017-02868-w 28596555 PMC5465099

[B34] Morisset D. Berjeaud J. M. Marion D. Lacombe C. Frère J. (2004). Mutational analysis of mesentericin Y105, an anti-listeria bacteriocin, for determination of impact on bactericidal activity, in vitro secondary structure, and membrane interaction. *Appl. Environ. Microbiol.* 70 4672–4680. 10.1128/AEM.70.8.4672-4680.2004 15294801 PMC492324

[B35] Paludan-Muller C. Madsen M. Sophanodora P. Gram L. Moller P. L. (2002a). Fermentation and microflora of plaa-som, a thai fermented fish product prepared with different salt concentrations. *Int. J. Food Microbiol.* 73 61–70. 10.1016/S0168-1605(01)00688-2 11883675

[B36] Paludan-Muller C. Valyasevi R. Huss H. H. Gram L. (2002b). Genotypic and phenotypic characterization of garlic-fermenting lactic acid bacteria isolated from som-fak, a Thai low-salt fermented fish product. *J. Appl. Microbiol.* 92 307–314. 10.1016/S0168-1605(98)00204-9 11849359

[B37] Peng C. Borges S. Magalhães R. Carvalheira A. Ferreira V. Casquete R. (2017). Characterization of anti-listerial bacteriocin produced by lactic acid bacteria isolated from traditional fermented foods from Cambodia. *Int. Food Res J.* 24 386–393.

[B38] Perez R. H. Zendo T. Sonomoto K. (2014). Novel bacteriocins from lactic acid bacteria (LAB): various structures and applications. *Microb. Cell Fact.* 13 1–13. 10.1186/1475-2859-13-S1-S3 25186038 PMC4155820

[B39] Prudêncio C. V. Mantovani H. C. Cecon P. R. Vanetti M. C. (2015). Differences in the antibacterial activity of nisin and bovicin HC5 against salmonella typhimurium under different temperature and pH conditions. *J. Appl. Microbiol.* 118 18–26. 10.1111/jam.12680 25358073

[B40] van Reenen C. A. Dicks L. M. Chikindas M. L. (2010). Isolation, purification and partial characterization of plantaricin 423, a bacteriocin produced by *Lactobacillus plantarum*. *J. Appl. Microbiol.* 84 1131–1137. 10.1046/j.1365-2672.1998.00451.x 9717299

[B41] Vos P. Garrity G. Jones D. Krieg N. R. Ludwig W. Rainey F. A. (2009). *Bergey’s Manual^®^ of Systematic Bacteriology*. New York, NY: Springer 10.5962/bhl.title.10728

[B42] Rojo-Bezares B. Saenz Y. Navarro L. Zarazaga M. Ruiz-Larrea F. Torres C. (2007). Coculture-inducible bacteriocin activity of *Lactobacillus plantarum* strain J23 isolated from grape must. *Food Microbiol.* 24 482–491. 10.1016/j.fm.2006.09.003 17367681

[B43] Snyder A. B. Worobo R. W. (2014). Chemical and genetic characterization of bacteriocins: antimicrobial peptides for food safety. *J. Sci. Food Agric.* 94 28–44. 10.1002/jsfa.6293 23818338

[B44] Sonsa-Ard N. Rodtong S. Chikindas M. L. Yongsawatdigul J. (2015). Characterization of bacteriocin produced by *Enterococcus faecium* CN-25 isolated from traditionally Thai fermented fish roe. *Food Control* 54(Suppl. C), 308–316. 10.1016/j.foodcont.2015.02.010

[B45] Tagg J. R. Dajani A. S. Wannamaker L. W. (1995). Bacteriocins of gram-positive bacteria. *Bacteriol. Rev.* 59 171–200. 10.1128/br.40.3.722-756.1976 PMC413978 791239

[B46] Vaughan A. O’Sullivan T. Van Sinderen D. (2012). Enhancing the microbiological stability of malt and beer &mdash; a review. *J. Inst. Brew.* 111 355–371. 10.1002/j.2050-0416.2005.tb00221.x 19714985

[B47] Wang Y. Shang N. Qin Y. Zhang Y. Zhang J. Li P. (2018). The complete genome sequence of *Lactobacillus plantarum* LPL-1, a novel antibacterial probiotic producing class IIa bacteriocin. *J. Biotechnol.* 266 84–88. 10.1016/j.jbiotec.2017.12.006 29229543

[B48] Wen L. S. Philip K. Ajam N. (2016). Purification, characterization and mode of action of plantaricin K25 produced by *Lactobacillus plantarum*. *Food Control* 60(Suppl. C), 430–439. 10.1016/j.foodcont.2015.08.010

[B49] Yang S.-C. Lin C.-H. Sung C. T. Fang J.-Y. (2014). Antibacterial activities of bacteriocins: application in foods and pharmaceuticals. *Front. Microbiol.* 5:241 10.3389/fmicb.2014.00241 PMC4033612 24904554

[B50] Yi L. Dang J. Zhang L. Wu Y. Liu B. Xin L. (2016). Purification, characterization and bactericidal mechanism of a broad spectrum bacteriocin with antimicrobial activity against multidrug-resistant strains produced by *Lactobacillus coryniformis* XN8. *Food Control* 67 53–62. 10.1016/j.foodcont.2016.02.008

[B51] Zhao L. Wang S. Liu F. Dong P. (2013). Comparing the effect of high hydrostatic pressure and thermal pasteurization combined with nisin on the quality of cucumber juice drinks. *Innov. Food Sci. Emerg. Technol. IFSET* 17 27–36. 10.1016/j.ifset.2012.10.004

[B52] Zhao S. Han J. Bie X. Lu Z. Zhang C. Lv F. (2016). Purification and characterization of plantaricin JLA-9: a novel bacteriocin against *Bacillus* spp. produced by *Lactobacillus plantarum JLA*-9 from Suan-Tsai, a traditional chinese fermented cabbage. *J. Agric. Food Chem.* 64 2754–2764. 10.1021/acs.jafc.5b05717 26985692

[B53] Zhu X. Zhao Y. Sun Y. Gu Q. (2014). Purification and characterisation of plantaricin ZJ008, a novel bacteriocin against *Staphylococcus* spp. from *Lactobacillus plantarum* ZJ008. *Food Chem.* 165 216–223. 10.1016/j.foodchem.2014.05.034 25038669

