# Supplementary figures and images for: Purification and Characterization of Plantaricin LPL-1, a Novel Class IIa Bacteriocin Produced by Lactobacillus plantarum LPL-1 Isolated From Fermented Fish
Source: Front Microbiol. 2018 Sep 28;9:2276. doi: 10.3389/fmicb.2018.02276 (PMC6172451; doi:10.3389/fmicb.2018.02276)

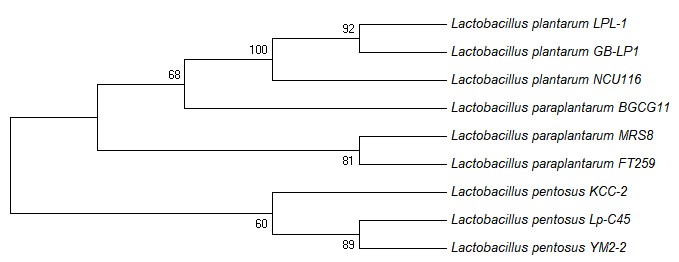

Supplement: FIGURE S1 — Phylogenetic tree of L. plantarum LPL-1 according to 16S rRNA sequence. [file Image_1.JPEG]
