# Supplementary material for: Purification and Characterization of Plantaricin LPL-1, a Novel Class IIa Bacteriocin Produced by Lactobacillus plantarum LPL-1 Isolated From Fermented Fish
Source: Front Microbiol. 2018 Sep 28;9:2276. doi: 10.3389/fmicb.2018.02276 (PMC6172451; doi:10.3389/fmicb.2018.02276)
Supplement: TABLE S1 — Antibacterial activity of screened strains in primary screening process. [file Table_1.DOC]

**TABLE S1** Antibacterial activity of screened strains in primary screening process

| Screened strains | Diameter of inhibition (mm) |
| --- | --- |
| LPL-1 | 12.34±0.18 |
| LPL-2 | 8.48±0.14 |
| LPL-3 | 7.95±0.12 |
| LPL-4 | 8.01±0.16 |
| LPL-5 | 8.55±0.13 |
| LPL-6 | 10.43±0.21 |
| LPL-7 | 9.45±0.14 |
| LPL-8 | 9.65±0.23 |
| LPL-9 | 10.01±0.17 |
| LPL-10 | 10.46±0.22 |
| LPL-11 | 7.98±0.15 |
| LPL-12 | 8.49±0.18 |
| LPL-13 | 7.92±0.16 |
| LPL-14 | 9.32±0.21 |
| LPL-15 | 9.87±0.19 |
| LPL-16 | 11.67±0.24 |
| LPL-17 | 10.22±0.13 |
| LPL-18 | 9.56±0.12 |
| LPL-19 | 9.77±0.15 |
| LPL-20 | 8.65±0.19 |
| LPL-21 | 10.39±0.12 |
| LPL-22 | 9.16±0.22 |
| LPL-23 | 8.97±0.14 |
| LPL-24 | 9.94±0.19 |
| LPL-25 | 11.98±0.15 |
| LPL-26 | 9.68±0.13 |
| LPL-27 | 8.98±0.16 |
| LPL-28 | 10.55±0.16 |
| LPL-29 | 9.69±0.24 |
| LPL-30 | 10.02±0.21 |
| LPL-31 | 10.51±0.22 |
