# Supplementary material for: Purification and Characterization of Plantaricin LPL-1, a Novel Class IIa Bacteriocin Produced by Lactobacillus plantarum LPL-1 Isolated From Fermented Fish
Source: Front Microbiol. 2018 Sep 28;9:2276. doi: 10.3389/fmicb.2018.02276 (PMC6172451; doi:10.3389/fmicb.2018.02276)
Supplement: TABLE S2 — 16S rRNA sequence alignment of Lactobacillus plantarum LPL-1 from NCBI database. [file Table_2.doc]

Table S2. 16S rRNA sequence alignment of *Lactobacillus plantarum* LPL-1from NCBI database.

>CP021997.1 Lactobacillus plantarum strain LPL-1, complete genome

TTAATTTGAGAGTTTGATCCTGGCTCAGGACGAACGCTGGCGGCGTGCCTAATACATGCAAGTCGAACGAACTCTGGTAT

TGATTGGTGCTTGCATCATGATTTGCATTTGAGTGAGTGGCGAACTGGTGAGTAACACGTGGGAAACCTGCCCAGAAGCG

GGGGATAACACCTGGAAACAGATGCTAATACCGCATAACAACTTGGACCGCATGGTCCGAGTTTGAAAGATGGCTTCGGC

TATCACTTTTGGATGGTCCCGCGGCGTATTAGCTAGATGGTGAGGTAACGGCTCACCATGGCAATGATACGTAGCCGACC

TGAGAGGGTAATCGGCCACATTGGGACTGAGACACGGCCCAAACTCCTACGGGAGGCAGCAGTAGGGAATCTTCCACAAT

GGACGAAAGTCTGATGGAGCAACGCCGCGTGAGTGAAGAAGGGTTTCGACTCGTAAAACTCTGTTGTTAAAGAAGAACAT

ATCTGAGAGTAACTGTTCAGGTATTGACGGTATTTAACCAGAAAGCCACGGCTAACTACGTGCCAGCAGCCGCGGTAATA

CGTAGGTGGCAAGCGTTGTCCGGATTTATTGGGCGTAAAGCGAGCGCAGGCGGTTTTTTAAGTCTGATGTGAAAGCCTTC

GGCTCAACCGAAGAAGTGCATCGGAAACTGGGAAACTTGAGTGCAGAAGAGGACAGTGGAACTCCATGTGTAGCGGTGAA

ATGCGTAGATATATGGAAGAACACCAGTGGCGAAGGCGGCTGTCTGGTCTGTAACTGACGCTGAGGCTCGAAAGTATGGG

TAGCAAACAGGATTAGATACCCTGGTAGTCCATACCGTAAACGATGAATGCTAAGTGTTGGAGGGTTTCCGCCCTTCAGT

GCTGCAGCTAACGCATTAAGCATTCCGCCTGGGGAGTACGGCCGCAAGGCTGAAACTCAAAGGAATTGACGGGGGCCCGC

ACAAGCGGTGGAGCATGTGGTTTAATTCGAAGCTACGCGAAGAACCTTACCAGGTCTTGACATACTATGCAAATCTAAGA

GATTAGACGTTCCCTTCGGGGACATGGATACAGGTGGTGCATGGTTGTCGTCAGCTCGTGTCGTGAGATGTTGGGTTAAG

TCCCGCAACGAGCGCAACCCTTATTATCAGTTGCCAGCATTAAGTTGGGCACTCTGGTGAGACTGCCGGTGACAAACCGG

AGGAAGGTGGGGATGACGTCAAATCATCATGCCCCTTATGACCTGGGCTACACACGTGCTACAATGGATGGTACAACGAG

TTGCGAACTCGCGAGAGTAAGCTAATCTCTTAAAGCCATTCTCAGTTCGGATTGTAGGCTGCAACTCGCCTACATGAAGT

CGGAATCGCTAGTAATCGCGGATCAGCATGCCGCGGTGAATACGTTCCCGGGCCTTGTACACACCGCCCGTCACACCATG

AGAGTTTGTAACACCCAAAGTCGGTGGGGTAACCTTTTAGGAACCAGCCGCCTAAGGTGGGACAGATGATTAGGGTGAAG

TCGTAACAAGGTAGCCGTAGGAGAACCTGCGGCTGGATCACCTCCTTTCTAAGGAA

>CP021997.1 Lactobacillus plantarum strain LPL-1, complete genome

TTCCTTAGAAAGGAGGTGATCCAGCCGCAGGTTCTCCTACGGCTACCTTGTTACGACTTCACCCTAATCATCTGTCCCAC

CTTAGGCGGCTGGTTCCTAAAAGGTTACCCCACCGACTTTGGGTGTTACAAACTCTCATGGTGTGACGGGCGGTGTGTAC

AAGGCCCGGGAACGTATTCACCGCGGCATGCTGATCCGCGATTACTAGCGATTCCGACTTCATGTAGGCGAGTTGCAGCC

TACAATCCGAACTGAGAATGGCTTTAAGAGATTAGCTTACTCTCGCGAGTTCGCAACTCGTTGTACCATCCATTGTAGCA

CGTGTGTAGCCCAGGTCATAAGGGGCATGATGATTTGACGTCATCCCCACCTTCCTCCGGTTTGTCACCGGCAGTCTCAC

CAGAGTGCCCAACTTAATGCTGGCAACTGATAATAAGGGTTGCGCTCGTTGCGGGACTTAACCCAACATCTCACGACACG

AGCTGACGACAACCATGCACCACCTGTATCCATGTCCCCGAAGGGAACGTCTAATCTCTTAGATTTGCATAGTATGTCAA

GACCTGGTAAGGTTCTTCGCGTAGCTTCGAATTAAACCACATGCTCCACCGCTTGTGCGGGCCCCCGTCAATTCCTTTGA

GTTTCAGCCTTGCGGCCGTACTCCCCAGGCGGAATGCTTAATGCGTTAGCTGCAGCACTGAAGGGCGGAAACCCTCCAAC

ACTTAGCATTCATCGTTTACGGTATGGACTACCAGGGTATCTAATCCTGTTTGCTACCCATACTTTCGAGCCTCAGCGTC

AGTTACAGACCAGACAGCCGCCTTCGCCACTGGTGTTCTTCCATATATCTACGCATTTCACCGCTACACATGGAGTTCCA

CTGTCCTCTTCTGCACTCAAGTTTCCCAGTTTCCGATGCACTTCTTCGGTTGAGCCGAAGGCTTTCACATCAGACTTAAA

AAACCGCCTGCGCTCGCTTTACGCCCAATAAATCCGGACAACGCTTGCCACCTACGTATTACCGCGGCTGCTGGCACGTA

GTTAGCCGTGGCTTTCTGGTTAAATACCGTCAATACCTGAACAGTTACTCTCAGATATGTTCTTCTTTAACAACAGAGTT

TTACGAGCCGAAACCCTTCTTCACTCACGCGGCGTTGCTCCATCAGACTTTCGTCCATTGTGGAAGATTCCCTACTGCTG

CCTCCCGTAGGAGTTTGGGCCGTGTCTCAGTCCCAATGTGGCCGATTACCCTCTCAGGTCGGCTACGTATCATTGCCATG

GTGAGCCGTTACCTCACCATCTAGCTAATACGCCGCGGGACCATCCAAAAGTGATAGCCGAAGCCATCTTTCAAACTCGG

ACCATGCGGTCCAAGTTGTTATGCGGTATTAGCATCTGTTTCCAGGTGTTATCCCCCGCTTCTGGGCAGGTTTCCCACGT

GTTACTCACCAGTTCGCCACTCACTCAAATGTAAATCATGATGCAAGCACCAATCAATACCAGAGTTCGTTCGACTTGCA

TGTATTAGGCACGCCGCCAGCGTTCGTCCTGAGCCAGGATCAAACTCTCAAATTAA

>CP021997.1 Lactobacillus plantarum strain LPL-1, complete genome

TTAATTTGAGAGTTTGATCCTGGCTCAGGACGAACGCTGGCGGCGTGCCTAATACATGCAAGTCGAACGAACTCTGGTAT

TGATTGGTGCTTGCATCATGATTTACATTTGAGTGAGTGGCGAACTGGTGAGTAACACGTGGGAAACCTGCCCAGAAGCG

GGGGATAACACCTGGAAACAGATGCTAATACCGCATAACAACTTGGACCGCATGGTCCGAGTTTGAAAGATGGCTTCGGC

TATCACTTTTGGATGGTCCCGCGGCGTATTAGCTAGATGGTGAGGTAACGGCTCACCATGGCAATGATACGTAGCCGACC

TGAGAGGGTAATCGGCCACATTGGGACTGAGACACGGCCCAAACTCCTACGGGAGGCAGCAGTAGGGAATCTTCCACAAT

GGACGAAAGTCTGATGGAGCAACGCCGCGTGAGTGAAGAAGGGTTTCGGCTCGTAAAACTCTGTTGTTAAAGAAGAACAT

ATCTGAGAGTAACTGTTCAGGTATTGACGGTATTTAACCAGAAAGCCACGGCTAACTACGTGCCAGCAGCCGCGGTAATA

CGTAGGTGGCAAGCGTTGTCCGGATTTATTGGGCGTAAAGCGAGCGCAGGCGGTTTTTTAAGTCTGATGTGAAAGCCTTC

GGCTCAACCGAAGAAGTGCATCGGAAACTGGGGAACTTGAGTGCAGAAGAGGACAGTGGAACTCCATGTGTAGCGGTGAA

ATGCGTAGATATATGGAAGAACACCAGTGGCGAAGGCGGCTGTCTGGTCTGTAACTGACGCTGAGGCTCGAAAGTATGGG

TAGCAAACAGGATTAGATACCCTGGTAGTCCATACCGTAAACGATGAATGCTAAGTGTTGGAGGGTTTCCGCCCTTCAGT

GCTGCAGCTAACGCATTAAGCATTCCGCCTGGGGAGTACGGCCGCAAGGCTGAAACTCAAAGGAATTGACGGGGGCCCGC

ACAAGCGGTGGAGCATGTGGTTTAATTCGAAGCTACGCGAAGAACCTTACCAGGTCTTGACATACTATGCAAATCTAAGA

GATTAGACGTTCCCTTCGGGGACATGGATACAGGTGGTGCATGGTTGTCGTCAGCTCGTGTCGTGAGATGTTGGGTTAAG

TCCCGCAACGAGCGCAACCCTTATTATCAGTTGCCAGCATTAAGTTGGGCACTCTGGTGAGACTGCCGGTGACAAACCGG

AGGAAGGTGGGGATGACGTCAAATCATCATGCCCCTTATGACCTGGGCTACACACGTGCTACAATGGATGGTACAACGAG

TTGCGAACTCGCGAGAGTAAGCTAATCTCTTAAAGCCATTCTCAGTTCGGATTGTAGGCTGCAACTCGCCTACATGAAGT

CGGAATCGCTAGTAATCGCGGATCAGCATGCCGCGGTGAATACGTTCCCGGGCCTTGTACACACCGCCCGTCACACCATG

AGAGTTTGTAACACCCAAAGTCGGTGGGGTAACCTTTTAGGAACCAGCCGCCTAAGGTGGGACAGATGATTAGGGTGAAG

TCGTAACAAGGTAGCCGTAGGAGAACCTGCGGCTGGATCACCTCCTTTCTAAGGAA

>CP021997.1 Lactobacillus plantarum strain LPL-1, complete genome

TTAATTTGAGAGTTTGATCCTGGCTCAGGACGAACGCTGGCGGCGTGCCTAATACATGCAAGTCGAACGAACTCTGGTAT

TGATTGGTGCTTGCATCATGATTTACATTTGAGTGAGTGGCGAACTGGTGAGTAACACGTGGGAAACCTGCCCAGAAGCG

GGGGATAACACCTGGAAACAGATGCTAATACCGCATAACAACTTGGACCGCATGGTCCGAGTTTGAAAGATGGCTTCGGC

TATCACTTTTGGATGGTCCCGCGGCGTATTAGCTAGATGGTGAGGCAACGGCTCACCATGGCAATGATACGTAGCCGACC

TGAGAGGGTAATCGGCCACATTGGGACTGAGACACGGCCCAAACTCCTACGGGAGGCAGCAGTAGGGAATCTTCCACAAT

GGACGAAAGTCTGATGGAGCAACGCCGCGTGAGTGAAGAAGGGTTTCGGCTCGTAAAACTCTGTTGTTAAAGAAGAACAT

ATCTGAGAGTAACTGTTCAGGTATTGACGGTATTTAACCAGAAAGCCACGGCTAACTACGTGCCAGCAGCCGCGGTAATA

CGTAGGTGGCAAGCGTTGTCCGGATTTATTGGGCGTAAAGCGAGCGCAGGCGGTTTTTTAAGTCTGATGTGAAAGCCTTC

GGCTCAACCGAAGAAGTGCATCGGAAACTGGGAAACTTGAGTGCAGAAGAGGACAGTGGAACTCCATGTGTAGCGGTGAA

ATGCGTAGATATATGGAAGAACACCAGTGGCGAAGGCGGCTGTCTGGTCTGTAACTGACGCTGAGGCTCGAAAGTATGGG

TAGCAAACAGGATTAGATACCCTGGTAGTCCATACCGTAAACGATGAATGCTAAGTGTTGGAGGGTTTCCGCCCTTCAGT

GCTGCAGCTAACGCATTAAGCATTCCGCCTGGGGAGTACGGCCGCAAGGCTGAAACTCAAAGGAATTGACGGGGGCCCGC

ACAAGCGGTGGAGCATGTGGTTTAATTCGAAGCTACGCGAAGAACCTTACCAGGTCTTGACATACTATGCAAATCTAAGA

GATTAGACGTTCCCTTCGGGGACATGGATACAGGTGGTGCATGGTTGTCGTCAGCTCGTGTCGTGAGATGTTGGGTTAAG

TCCCGCAACGAGCGCAACCCTTATTATCAGTTGCCAGCATTAAGTTGGGCACTCTGGTGAGACTGCCGGTGACAAACCGG

AGGAAGGTGGGGATGACGTCAAATCATCATGCCCCTTATGACCTGGGCTACACACGTGCTACAATGGATGGTACAACGAG

TTGCGAACTCGCGAGAGTAAGCTAATCTCTTAAAGCCATTCTCAGTTCGGATTGTAGGCTGCAACTCGCCTACATGAAGT

CGGAATCGCTAGTAATCGCGGATCAGCATGCCGCGGTGAATACGTTCCCGGGCCTTGTACACACCGCCCGTCACACCATG

AGAGTTTGTAACACCCAAAGTCGGTGGGGTAACCTTTTAGGAACCAGCCGCCTAAGGTGGGACAGATGATTAGGGTGAAG

TCGTAACAAGGTAGCCGTAGGAGAACCTGCGGCTGGATCACCTCCTTTCTAAGGAA

>CP021997.1 Lactobacillus plantarum strain LPL-1, complete genome

TTCCTTAGAAAGGAGGTGATCCAGCCGCAGGTTCTCCTACGGCTACCTTGTTACGACTTCACCCTAATCATCTGTCCCAC

CTTAGGCGGCTGGTTCCTAAAAGGTTACCCCACCGACTTTGGGTGTTACAAACTCTCATGGTGTGACGGGCGGTGTGTAC

AAGGCCCGGGAACGTATTCACCGCGGCATGCTGATCCGCGATTACTAGCGATTCCGACTTCATGTAGGCGAGTTGCAGCC

TACAATCCGAACTGAGAATGGCTTTAAGAGATTAGCTTACTCTCGCGAGTTCGCAACTCGTTGTACCATCCATTGTAGCA

CGTGTGTAGCCCAGGTCATAAGGGGCATGATGATTTGACGTCATCCCCACCTTCCTCCGGTTTGTCACCGGCAGTCTCAC

CAGAGTGCCCAACTTAATGCTGGCAACTGATAATAAGGGTTGCGCTCGTTGCGGGACTTAACCCAACATCTCACGACACG

AGCTGACGACAACCATGCACCACCTGTATCCATGTCCCCGAAGGGAACGTCTAATCTCTTAGATTTGCATAGTATGTCAA

GACCTGGTAAGGTTCTTCGCGTAGCTTCGAATTAAACCACATGCTCCACCGCTTGTGCGGGCCCCCGTCAATTCCTTTGA

GTTTCAGCCTTGCGGCCGTACTCCCCAGGCGGAATGCTTAATGCGTTAGCTGCAGCACTGAAGGGCGGAAACCCTCCAAC

ACTTAGCATTCATCGTTTACGGTATGGACTACCAGGGTATCTAATCCTGTTTGCTACCCATACTTTCGAGCCTCAGCGTC

AGTTACAGACCAGACAGCCGCCTTCGCCACTGGTGTTCTTCCATATATCTACGCATTTCACCGCTACACATGGAGTTCCA

CTGTCCTCTTCTGCACTCAAGTTTCCCAGTTTCCGATGCACTTCTTCGGTTGAGCCGAAGGCTTTCACATCAGACTTAAA

AAACCGCCTGCGCTCGCTTTACGCCCAATAAATCCGGACAACGCTTGCCACCTACGTATTACCGCGGCTGCTGGCACGTA

GTTAGCCGTGGCTTTCTGGTTAAATACCGTCAATACCTGAACAGTTACTCTCAGATATGTTCTTCTTTAACAACAGAGTT

TTACGAGCCGAAACCCTTCTTCACTCACGCGGCGTTGCTCCATCAGACTTTCGTCCATTGTGGAAGATTCCCTACTGCTG

CCTCCCGTAGGAGTTTGGGCCGTGTCTCAGTCCCAATGTGGCCGATTACCCTCTCAGGTCGGCTACGTATCATTGCCATG

GTGAGCCGTTACCCCACCATCTAGCTAATACGCCGCGGGACCATCCAAAAGTGATAGCCGAAGCCATCTTTCAAGCTCGG

ACCATGCGGTCCAAGTTGTTATGCGGTATTAGCATCTGTTTCCAGGTGTTATCCCCCGCTTCTGGGCAGGTTTCCCACGT

GTTACTCACCAGTTCGCCACTCACTCAAATGTAAATCATGATGCAAGCACCAATCAATACCAGAGTTCGTTCGACTTGCA

TGTATTAGGCACGCCGCCAGCGTTCGTCCTGAGCCAGGATCAAACTCTCAAATTAA

>CP020564.1 Lactobacillus plantarum strain GB-LP1, complete genome

TTCCTTAGAAAGGAGGTGATCCAGCCGCAGGTTCTCCTACGGCTACCTTGTTACGACTTCACCCTAATCATCTGTCCCAC

CTTAGGCGGCTGGTTCCTAAAAGGTTACCCCACCGACTTTGGGTGTTACAAACTCTCATGGTGTGACGGGCGGTGTGTAC

AAGGCCCGGGAACGTATTCACCGCGGCATGCTGATCCGCGATTACTAGCGATTCCGACTTCATGTAGGCGAGTTGCAGCC

TACAATCCGAACTGAGAATGGCTTTAAGAGATTAGCTTACTCTCGCGAGTTCGCAACTCGTTGTACCATCCATTGTAGCA

CGTGTGTAGCCCAGGTCATAAGGGGCATGATGATTTGACGTCATCCCCACCTTCCTCCGGTTTGTCACCGGCAGTCTCAC

CAGAGTGCCCAACTTAATGCTGGCAACTGATAATAAGGGTTGCGCTCGTTGCGGGACTTAACCCAACATCTCACGACACG

AGCTGACGACAACCATGCACCACCTGTATCCATGTCCCCGAAGGGAACGTCTAATCTCTTAGATTTGCATAGTATGTCAA

GACCTGGTAAGGTTCTTCGCGTAGCTTCGAATTAAACCACATGCTCCACCGCTTGTGCGGGCCCCCGTCAATTCCTTTGA

GTTTCAGCCTTGCGGCCGTACTCCCCAGGCGGAATGCTTAATGCGTTAGCTGCAGCACTGAAGGGCGGAAACCCTCCAAC

ACTTAGCATTCATCGTTTACGGTATGGACTACCAGGGTATCTAATCCTGTTTGCTACCCATACTTTCGAGCCTCAGCGTC

AGTTACAGACCAGACAGCCGCCTTCGCCACTGGTGTTCTTCCATATATCTACGCATTTCACCGCTACACATGGAGTTCCA

CTGTCCTCTTCTGCACTCAAGTTTCCCAGTTTCCGATGCACTTCTTCGGTTGAGCCGAAGGCTTTCACATCAGACTTAAA

AAACCGCCTGCGCTCGCTTTACGCCCAATAAATCCGGACAACGCTTGCCACCTACGTATTACCGCGGCTGCTGGCACGTA

GTTAGCCGTGGCTTTCTGGTTAAATACCGTCAATACCTGAACAGTTACTCTCAGATATGTTCTTCTTTAACAACAGAGTT

TTACGAGCCGAAACCCTTCTTCACTCACGCGGCGTTGCTCCATCAGACTTTCGTCCATTGTGGAAGATTCCCTACTGCTG

CCTCCCGTAGGAGTTTGGGCCGTGTCTCAGTCCCAATGTGGCCGATTACCCTCTCAGGTCGGCTACGTATCATTGCCATG

GTGAGCCGTTACCTCACCATCTAGCTAATACGCCGCGGGACCATCCAAAAGTGATAGCCGAAGCCATCTTTCAAACTCGG

ACCATGCGGTCCAAGTTGTTATGCGGTATTAGCATCTGTTTCCAGGTGTTATCCCCCGCTTCTGGGCAGGTTTCCCACGT

GTTACTCACCAGTTCGCCACTCACTCAAATGTAAATCATGATGCAAGCACCAATCAATACCAGAGTTCGTTCGACTTGCA

TGTATTAGGCACGCCGCCAGCGTTCGTCCTGAGCCAGGATCAAACTCTCAAATTAA

>CP020564.1 Lactobacillus plantarum strain GB-LP1, complete genome

TTAATTTGAGAGTTTGATCCTGGCTCAGGACGAACGCTGGCGGCGTGCCTAATACATGCAAGTCGAACGAACTCTGGTAT

TGATTGGTGCTTGCATCATGATTTACATTTGAGTGAGTGGCGAACTGGTGAGTAACACGTGGGAAACCTGCCCAGAAGCG

GGGGATAACACCTGGAAACAGATGCTAATACCGCATAACAACTTGGACCGCATGGTCCGAGTTTGAAAGATGGCTTCGGC

TATCACTTTTGGATGGTCCCGCGGCGTATTAGCTAGATGGTGGGGTAACGGCTCACCATGGCAATGATACGTAGCCGACC

TGAGAGGGTAATCGGCCACATTGGGACTGAGACACGGCCCAAACTCCTACGGGAGGCAGCAGTAGGGAATCTTCCACAAT

GGACGAAAGTCTGATGGAGCAACGCCGCGTGAGTGAAGAAGGGTTTCGGCTCGTAAAACTCTGTTGTTAAAGAAGAACAT

ATCTGAGAGTAACTGTTCAGGTATTGACGGTATTTAACCAGAAAGCCACGGCTAACTACGTGCCAGCAGCCGCGGTAATA

CGTAGGTGGCAAGCGTTGTCCGGATTTATTGGGCGTAAAGCGAGCGCAGGCGGTTTTTTAAGTCTGATGTGAAAGCCTTC

GGCTCAACCGAAGAAGTGCATCGGAAACTGGGAAACTTGAGTGCAGAAGAGGACAGTGGAACTCCATGTGTAGCGGTGAA

ATGCGTAGATATATGGAAGAACACCAGTGGCGAAGGCGGCTGTCTGGTCTGTAACTGACGCTGAGGCTCGAAAGTATGGG

TAGCAAACAGGATTAGATACCCTGGTAGTCCATACCGTAAACGATGAATGCTAAGTGTTGGAGGGTTTCCGCCCTTCAGT

GCTGCAGCTAACGCATTAAGCATTCCGCCTGGGGAGTACGGCCGCAAGGCTGAAACTCAAAGGAATTGACGGGGGCCCGC

ACAAGCGGTGGAGCATGTGGTTTAATTCGAAGCTACGCGAAGAACCTTACCAGGTCTTGACATACTATGCAAATCTAAGA

GATTAGACGTTCCCTTCGGGGACATGGATACAGGTGGTGCATGGTTGTCGTCAGCTCGTGTCGTGAGATGTTGGGTTAAG

TCCCGCAACGAGCGCAACCCTTATTATCAGTTGCCAGCATTAAGTTGGGCACTCTGGTGAGACTGCCGGTGACAAACCGG

AGGAAGGTGGGGATGACGTCAAATCATCATGCCCCTTATGACCTGGGCTACACACGTGCTACAATGGATGGTACAACGAG

TTGCGAACTCGCGAGAGTAAGCTAATCTCTTAAAGCCATTCTCAGTTCGGATTGTAGGCTGCAACTCGCCTACATGAAGT

CGGAATCGCTAGTAATCGCGGATCAGCATGCCGCGGTGAATACGTTCCCGGGCCTTGTACACACCGCCCGTCACACCATG

AGAGTTTGTAACACCCAAAGTCGGTGGGGTAACCTTTTAGGAACCAGCCGCCTAAGGTGGGACAGATGATTAGGGTGAAG

TCGTAACAAGGTAGCCGTAGGAGAACCTGCGGCTGGATCACCTCCTTTCTAAGGAA

>CP020564.1 Lactobacillus plantarum strain GB-LP1, complete genome

TTCCTTAGAAAGGAGGTGATCCAGCCGCAGGTTCTCCTACGGCTACCTTGTTACGACTTCACCCTAATCATCTGTCCCAC

CTTAGGCGGCTGGTTCCTAAAAGGTTACCCCACCGACTTTGGGTGTTACAAACTCTCATGGTGTGACGGGCGGTGTGTAC

AAGGCCCGGGAACGTATTCACCGCGGCATGCTGATCCGCGATTACTAGCGATTCCGACTTCATGTAGGCGAGTTGCAGCC

TACAATCCGAACTGAGAATGGCTTTAAGAGATTAGCTTACTCTCGCGAGTTCGCAACTCGTTGTACCATCCATTGTAGCA

CGTGTGTAGCCCAGGTCATAAGGGGCATGATGATTTGACGTCATCCCCACCTTCCTCCGGTTTGTCACCGGCAGTCTCAC

CAGAGTGCCCAACTTAATGCTGGCAACTGATAATAAGGGTTGCGCTCGTTGCGGGACTTAACCCAACATCTCACGACACG

AGCTGACGACAACCATGCACCACCTGTATCCATGTCCCCGAAGGGAACGTCTAATCTCTTAGATTTGCATAGTATGTCAA

GACCTGGTAAGGTTCTTCGCGTAGCTTCGAATTAAACCACATGCTCCACCGCTTGTGCGGGCCCCCGTCAATTCCTTTGA

GTTTCAGCCTTGCGGCCGTACTCCCCAGGCGGAATGCTTAATGCGTTAGCTGCAGCACTGAAGGGCGGAAACCCTCCAAC

ACTTAGCATTCATCGTTTACGGTATGGACTACCAGGGTATCTAATCCTGTTTGCTACCCATACTTTCGAGCCTCAGCGTC

AGTTACAGACCAGACAGCCGCCTTCGCCACTGGTGTTCTTCCATATATCTACGCATTTCACCGCTACACATGGAGTTCCA

CTGTCCTCTTCTGCACTCAAGTTTCCCAGTTTCCGATGCACTTCTTCGGTTGAGCCGAAGGCTTTCACATCAGACTTAAA

AAACCGCCTGCGCTCGCTTTACGCCCAATAAATCCGGACAACGCTTGCCACCTACGTATTACCGCGGCTGCTGGCACGTA

GTTAGCCGTGGCTTTCTGGTTAAATACCGTCAATACCTGAACAGTTACTCTCAGATATGTTCTTCTTTAACAACAGAGTT

TTACGAGCCGAAACCCTTCTTCACTCACGCGGCGTTGCTCCATCAGACTTTCGTCCATTGTGGAAGATTCCCTACTGCTG

CCTCCCGTAGGAGTTTGGGCCGTGTCTCAGTCCCAATGTGGCCGATTACCCTCTCAGGTCGGCTACGTATCATTGCCATG

GTGAGCCGTTGCCTCACCATCTAGCTAATACGCCGCGGGACCATCCAAAAGTGATAGCCGAAGCCATCTTTCAAACTCGG

ACCATGCGGTCCAAGTTGTTATGCGGTATTAGCATCTGTTTCCAGGTGTTATCCCCCACTTCTGGGCAGGTTTCCCACGT

GTTACTCACCAGTTCGCCACTCACTCAAATGTAAATCATGATGCAAGCACCAATCAATACCAGAGTTCGTTCGACTTGCA

TGTATTAGGCACGCCGCCAGCGTTCGTCCTGAGCCAGGATCAAACTCTCAAATTAA

>CP020564.1 Lactobacillus plantarum strain GB-LP1, complete genome

TTCCTTAGAAAGGAGGTGATCCAGCCGCAGGTTCTCCTACGGCTACCTTGTTACGACTTCACCCTAATCATCTGTCCCAC

CTTAGGCGGCTGGTTCCTAAAAGGTTACCCCACCGACTTTGGGTGTTACAAACTCTCATGGTGTGACGGGCGGTGTGTAC

AAGGCCCGGGAACGTATTCACCGCGGCATGCTGATCCGCGATTACTAGCGATTCCGACTTCATGTAGGCGAGTTGCAGCC

TACAATCCGAACTGAGAATGGCTTTAAGAGATTAGCTTACTCTCGCGAGTTCGCAACTCGTTGTACCATCCATTGTAGCA

CGTGTGTAGCCCAGGTCATAAGGGGCATGATGATTTGACGTCATCCCCACCTTCCTCCGGTTTGTCACCGGCAGTCTCAC

CAGAGTGCCCAACTTAATGCTGGCAACTGATAATAAGGGTTGCGCTCGTTGCGGGACTTAACCCAACATCTCACGACACG

AGCTGACGACAACCATGCACCACCTGTATCCATGTCCCCGAAGGGAACGTCTAATCTCTTAGATTTGCATAGTATGTCAA

GACCTGGTAAGGTTCTTCGCGTAGCTTCGAATTAAACCACATGCTCCACCGCTTGTGCGGGCCCCCGTCAATTCCTTTGA

GTTTCAGCCTTGCGGCCGTACTCCCCAGGCGGAATGCTTAATGCGTTAGCTGCAGCACTGAAGGGCGGAAACCCTCCAAC

ACTTAGCATTCATCGTTTACGGTATGGACTACCAGGGTATCTAATCCTGTTTGCTACCCATACTTTCGAGCCTCAGCGTC

AGTTACAGACCAGACAGCCGCCTTCGCCACTGGTGTTCTTCCATATATCTACGCATTTCACCGCTACACATGGAGTTCCA

CTGTCCTCTTCTGCACTCAAGTTTCCCAGTTTCCGATGCACTTCTTCGGTTGAGCCGAAGGCTTTCACATCAGACTTAAA

AAACCGCCTGCGCTCGCTTTACGCCCAATAAATCCGGACAACGCTTGCCACCTACGTATTACCGCGGCTGCTGGCACGTA

GTTAGCCGTGGCTTTCTGGTTAAATACCGTCAATACCTGAACAGTTACTCTCAGATATGTTCTTCTTTAACAACAGAGTT

TTACGAGCCGAAACCCTTCTTCACTCACGCGGCGTTGCTCCATCAGACTTTCGTCCATTGTGGAAGATTCCCTACTGCTG

CCTCCCGTAGGAGTTTGGGCCGTGTCTCAGTCCCAATGTGGCCGATTACCCTCTCAGGTCGGCTACGTATCATTGCCATG

GTGAGCCGTTACCCCACCATCTAGCTAATACGCCGCGGGACCATCCAAAAGTGATAGCCGAAGCCATCTTTCAAGCTCGG

ACCATGCGGTCCAAGTTGTTATGCGGTATTAGCATCTGTTTCCAGGTGTTATCCCCCACTTCTGGGCAGGTTTCCCACGT

GTTACTCACCAGTTCGCCACTCACTCAAATGTAAATCATGATGCAAGCACCAATCAATACCAGAGTTCGTTCGACTTGCA

TGTATTAGGCACGCCGCCAGCGTTCGTCCTGAGCCAGGATCAAACTCTCAAATTAA

>CP020564.1 Lactobacillus plantarum strain GB-LP1, complete genome

TTAATTTGAGAGTTTGATCCTGGCTCAGGACGAACGCTGGCGGCGTGCCTAATACATGCAAGTCGAACGAACTCTGGTAT

TGATTGGTGCTTGCATCATGATTTACATTTGAGTGAGTGGCGAACTGGTGAGTAACACGTGGGAAACCTGCCCAGAAGCG

GGGGATAACACCTGGAAACAGATGCTAATACCGCATAACAACTTGGACCGCATGGTCCGAGCTTGAAAGATGGCTTCAGC

TATCACTTTTGGATGGTCCCGCGGCGTATTAGCTAGATGGTGGGGTAACGGCTCACCATGGCAATGATACGTAGCCGACC

TGAGAGGGTAATCGGCCACATTGGGACTGAGACACGGCCCAAACTCCTACGGGAGGCAGCAGTAGGGAATCTTCCACAAT

GGACGAAAGTCTGATGGAGCAACGCCGCGTGAGTGAAGAAGGGTTTCGGCTCGTAAAACTCTGTTGTTAAAGAAGAACAT

ATCTGAGAGTAACTGTTCAGGTATTGACGGTATTTAACCAGAAAGCCACGGCTAACTACGTGCCAGCAGCCGCGGTAATA

CGTAGGTGGCAAGCGTTGTCCGGATTTATTGGGCGTAAAGCGAGCGCAGGCGGTTTTTTAAGTCTGATGTGAAAGCCTTC

GGCTCAACCGAAGAAGTGCATCGGAAACTGGGAGACTTGAGTGCAGAAGAGGACAGTGGAACTCCATGTGTAGCGGTGAA

ATGCGTAGATATATGGAAGAACACCAGTGGCGAAGGCGGCTGTCTGGTCTGTAACTGACGCTGAGGCTCGAAAGTATGGG

TAGCAAACAGGATTAGATACCCTGGTAGTCCATACCGTAAACGATGAATGCTAAGTGTTGGAGGGTTTCCGCCTTTCAGT

GCTGCAGCTAACGCATTAAGCATTCCGCCTGGGGAGTACGGCCGCAAGGCTGAAACTCAAAGGAATTGACGGGGGCCCGC

ACAAGCGGTGGAGCATGTGGTTTAATTCGAAGCTACGCGAAGAACCTTACCAGGTCTTGACATACTATGCAAATCTAAGA

GATTAGACGTTCCCTTCGGGGACATGGATACAGGTGGTGCATGGTTGTCGTCAGCTCGTGTCGTGAGATGTTGGGTTAAG

TCCCGCAACGAGCGCAACCCTTATTATCAGTTGCCAGCATTAAGTTGGGCACTCTGGTGAGACTGCCGGTGACAAACCGG

AGGAAGGTGGGGATGACGTCAAATCATCATGCCCCTTATGACCTGGGCTACACACGTGCTACAATGGATGGTACAACGAG

TTGCGAACTCGCGAGAGTAAGCTAATCTCTTAAAGCCATTCTCAGTTCGGATTGTAGGCTGCAACTCGCCTACATGAAGT

CGGAATCGCTAGTAATCGCGGATCAGCATGCCGCGGTGAATACGTTCCCGGGCCTTGTACACACCGCCCGTCACACCATG

AGAGTTTGTAACACCCAAAGTCGGTGGGGTAACCTTTTAGGAACCAGCCGCCTAAGGTGGGACAGATGATTAGGGTGAAG

TCGTAACAAGGTAGCCGTAGGAGAACCTGCGGCTGGATCACCTCCTTTCTAAGGAA

>CP016071.1 Lactobacillus plantarum strain NCU116, complete genome

TTAATTTGAGAGTTTGATCCTGGCTCAGGACGAACGCTGGCGGCGTGCCTAATACATGCAAGTCGAACGAACTCTGGTAT

TGATTGGTGCTTGCATCATGATTTACATTTGAGTGAGTGGCGAACTGGTGAGTAACACGTGGGAAACCTGCCCAGAAGCG

GGGGATAACACCTGGAAACAGATGCTAATACCGCATAACAACTTGGACCGCATGGTCCGAGTTTGAAAGATGGCTTCGGC

TATCACTTTTGGATGGTCCCGCGGCGTATTAGCTAGATGGTGAGGTAACGGCTCACCATGGCAATGATACGTAGCCGACC

TGAGAGGGTAATCGGCCACATTGGGACTGAGACACGGCCCAAACTCCTACGGGAGGCAGCAGTAGGGAATCTTCCACAAT

GGACGAAAGTCTGATGGAGCAACGCCGCGTGAGTGAAGAAGGGTTTCGGCTCGTAAAACTCTGTTGTTAAAGAAGAACAT

ATCTGAGAGTAACTGTTCAGGTATTGACGGTATTTAACCAGAAAGCCACGGCTAACTACGTGCCAGCAGCCGCGGTAATA

CGTAGGTGGCAAGCGTTGTCCGGATTTATTGGGCGTAAAGCGAGCGCAGGCGGTTTTTTAAGTCTGATGTGAAAGCCTTC

GGCTCAACCGAAGAAGTGCATCGGAAACTGGGGAACTTGAGTGCAGAAGAGGACAGTGGAACTCCATGTGTAGCGGTGAA

ATGCGTAGATATATGGAAGAACACCAGTGGCGAAGGCGGCTGTCTGGTCTGTAACTGACGCTGAGGCTCGAAAGTATGGG

TAGCAAACAGGATTAGATACCCTGGTAGTCCATACCGTAAACGATGAATGCTAAGTGTTGGAGGGTTTCCGCCCTTCAGT

GCTGCAGCTAACGCATTAAGCATTCCGCCTGGGGAGTACGGCCGCAAGGCTGAAACTCAAAGGAATTGACGGGGGCCCGC

ACAAGCGGTGGAGCATGTGGTTTAATTCGAAGCTACGCGAAGAACCTTACCAGGTCTTGACATACTATGCAAATCTAAGA

GATTAGACGTTCCCTTCGGGGACATGGATACAGGTGGTGCATGGTTGTCGTCAGCTCGTGTCGTGAGATGTTGGGTTAAG

TCCCGCAACGAGCGCAACCCTTATTATCAGTTGCCAGCATTAAGTTGGGCACTCTGGTGAGACTGCCGGTGACAAACCGG

AGGAAGGTGGGGATGACGTCAAATCATCATGCCCCTTATGACCTGGGCTACACACGTGCTACAATGGATGGTACAACGAG

TTGCGAACTCGCGAGAGTAAGCTAATCTCTTAAAGCCATTCTCAGTTCGGATTGTAGGCTGCAACTCGCCTACATGAAGT

CGGAATCGCTAGTAATCGCGGATCAGCATGCCGCGGTGAATACGTTCCCGGGCCTTGTACACACCGCCCGTCACACCATG

AGAGTTTGTAACACCCAAAGTCGGTGGGGTAACCTTTTAGGAACCAGCCGCCTAAGGTGGGACAGATGATTAGGGTGAAG

TCGTAACAAGGTAGCCGTAGGAGAACCTGCGGCTGGATCACCTCCTTTCTAAGGAA

>CP016071.1 Lactobacillus plantarum strain NCU116, complete genome

TTAATTTGAGAGTTTGATCCTGGCTCAGGACGAACGCTGGCGGCGTGCCTAATACATGCAAGTCGAACGAACTCTGGTAT

TGATTGGTGCTTGCATCATGATTTACATTTGAGTGAGTGGCGAACTGGTGAGTAACACGTGGGAAACCTGCCCAGAAGCG

GGGGATAACACCTGGAAACAGATGCTAATACCGCATAACAACTTGGACCGCATGGTCCGAGTTTGAAAGATGGCTTCGGC

TATCACTTTTGGATGGTCCCGCGGCGTATTAGCTAGATGGTGAGGTAACGGCTCACCATGGCAATGATACGTAGCCGACC

TGAGAGGGTAATCGGCCACATTGGGACTGAGACACGGCCCAAACTCCTACGGGAGGCAGCAGTAGGGAATCTTCCACAAT

GGACGAAAGTCTGATGGAGCAACGCCGCGTGAGTGAAGAAGGGTTTCGGCTCGTAAAACTCTGTTGTTAAAGAAGAACAT

ATCTGAGAGTAACTGTTCAGGTATTGACGGTATTTAACCAGAAAGCCACGGCTAACTACGTGCCAGCAGCCGCGGTAATA

CGTAGGTGGCAAGCGTTGTCCGGATTTATTGGGCGTAAAGCGAGCGCAGGCGGTTTTTTAAGTCTGATGTGAAAGCCTTC

GGCTCAACCGAAGAAGTGCATCGGAAACTGGGAAACTTGAGTGCAGAAGAGGACAGTGGAACTCCATGTGTAGCGGTGAA

ATGCGTAGATATATGGAAGAACACCAGTGGCGAAGGCGGCTGTCTGGTCTGTAACTGACGCTGAGGCTCGAAAGTATGGG

TAGCAAACAGGATTAGATACCCTGGTAGTCCATACCGTAAACGATGAATGCTAAGTGTTGGAGGGTTTCCGCCCTTCAGT

GCTGCAGCTAACGCATTAAGCATTCCGCCTGGGGAGTACGGCCGCAAGGCTGAAACTCAAAGGAATTGACGGGGGCCCGC

ACAAGCGGTGGAGCATGTGGTTTAATTCGAAGCTACGCGAAGAACCTTACCAGGTCTTGACATACTATGCAAATCTAAGA

GATTAGACGTTCCCTTTGGGGACATGGATACAGGTGGTGCATGGTTGTCGTCAGCTCGTGTCGTGAGATGTTGGGTTAAG

TCCCGCAACGAGCGCAACCCTTATTATCAGTTGCCAGCATTAAGTTGGGCACTCTGGTGAGACTGCCGGTGACAAACCGG

AGGAAGGTGGGGATGACGTCAAATCATCATGCCCCTTATGACCTGGGCTACACACGTGCTACAATGGATGGTACAACGAG

TTGCGAACTCGCGAGAGTAAGCTAATCTCTTAAAGCCATTCTCAGTTCGGATTGTAGGCTGCAACTCGCCTACATGAAGT

CGGAATCGCTAGTAATCGCGGATCAGCATGCCGCGGTGAATACGTTCCCGGGCCTTGTACACACCGCCCGTCACACCATG

AGAGTTTGTAACACCCAAAGTCGGTGGGGTAACCTTTTAGGAACCAGCCGCCTAAGGTGGGACAGATGATTAGGGTGAAG

TCGTAACAAGGTAGCCGTAGGAGAACCTGCGGCTGGATCACCTCCTTTCTAAGGAA

>CP016071.1 Lactobacillus plantarum strain NCU116, complete genome

TTAATTTGAGAGTTTGATCCTGGCTCAGGACGAACGCTGGCGGCGTGCCTAATACATGCAAGTCGAACGAACTCTGGTAT

TGATTGGTGCTTGCATCATGATTTACATTTGAGTGAGTGGCGAACTGGTGAGTAACACGTGGGAAACCTGCCCAGAAGCG

GGGGATAACACCTGGAAACAGATGCTAATACCGCATAACAACTTGGACCGCATGGTCCGAGTTTGAAAGATGGCTTCGGC

TATCACTTTTGGATGGTCCCGCGGCGTATTAGCTAGATGGTGGGGTAACGGCTCACCATGGCAATGATACGTAGCCGACC

TGAGAGGGTAATCGGCCACATTGGGACTGAGACACGGCCCAAACTCCTACGGGAGGCAGCAGTAGGGAATCTTCCACAAT

GGACGAAAGTCTGATGGAGCAACGCCGCGTGAGTGAAGAAGGGTTTCGGCTCGTAAAACTCTGTTGTTAAAGAAGAACAT

ATCTGAGAGTAACTGTTCAGGTATTGACGGTATTTAACCAGAAAGCCACGGCTAACTACGTGCCAGCAGCCGCGGTAATA

CGTAGGTGGCAAGCGTTGTCCGGATTTATTGGGCGTAAAGCGAGCGCAGGCGGTTTTTTAAGTCTGATGTGAAAGCCTTC

GGCTCAACCGAAGAAGTGCATCGGAAACTGGGAAACTTGAGTGCAGAAGAGGACAGTGGAACTCCATGTGTAGCGGTGAA

ATGCGTAGATATATGGAAGAACACCAGTGGCGAAGGCGGCTGTCTGGTCTGTAACTGACGCTGAGGCTCGAAAGTATGGG

TAGCAAACAGGATTAGATACCCTGGTAGTCCATACCGTAAACGATGAATGCTAAGTGTTGGAGGGTTTCCGCCCTTCAGT

GCTGCAGCTAACGCATTAAGCATTCCGCCTGGGGAGTACGGCCGCAAGGCTGAAACTCAAAGGAATTGACGGGGGCCCGC

ACAAGCGGTGGAGCATGTGGTTTAATTCGAAGCTACGCGAAGAACCTTACCAGGTCTTGACATACTATGCAAATCTAAGA

GATTAGACGTTCCCTTCGGGGACATGGATACAGGTGGTGCATGGTTGTCGTCAGCTCGTGTCGTGAGATGTTGGGTTAAG

TCCCGCAACGAGCGCAACCCTTATTATCAGTTGCCAGCATTAAGTTGGGCACTCTGGTGAGACTGCCGGTGACAAACCGG

AGGAAGGTGGGGATGACGTCAAATCATCATGCCCCTTATGACCTGGGCTACACACGTGCTACAATGGATGGTACAACGAG

TTGCGAACTCGCGAGAGTAAGCTAATCTCTTAAAGCCATTCTCAGTTCGGATTGTAGGCTGCAACTCGCCTACATGAAGT

CGGAATCGCTAGTAATCGCGGATCAGCATGCCGCGGTGAATACGTTCCCGGGCCTTGTACACACCGCCCGTCACACCATG

AGAGTTTGTAACACCCAAAGTCGGTGGGGTAACCTTTTAGGAACCAGCCGCCTAAGGTGGGACAGATGATTAGGGTGAAG

TCGTAACAAGGTAGCCGTAGGAGAACCTGCGGCTGGATCACCTCCTTTCTAAGGAA

>CP016071.1 Lactobacillus plantarum strain NCU116, complete genome

TTCCTTAGAAAGGAGGTGATCCAGCCGCAGGTTCTCCTACGGCTACCTTGTTACGACTTCACCCTAATCATCTGTCCCAC

CTTAGGCGGCTGGTTCCTAAAAGGTTACCCCACCGACTTTGGGTGTTACAAACTCTCATGGTGTGACGGGCGGTGTGTAC

AAGGCCCGGGAACGTATTCACCGCGGCATGCTGATCCGCGATTACTAGCGATTCCGACTTCATGTAGGCGAGTTGCAGCC

TACAATCCGAACTGAGAATGGCTTTAAGAGATTAGCTTACTCTCGCGAGTTCGCAACTCGTTGTACCATCCATTGTAGCA

CGTGTGTAGCCCAGGTCATAAGGGGCATGATGATTTGACGTCATCCCCACCTTCCTCCGGTTTGTCACCGGCAGTCTCAC

CAGAGTGCCCAACTTAATGCTGGCAACTGATAATAAGGGTTGCGCTCGTTGCGGGACTTAACCCAACATCTCACGACACG

AGCTGACGACAACCATGCACCACCTGTATCCATGTCCCCGAAGGGAACGTCTAATCTCTTAGATTTGCATAGTATGTCAA

GACCTGGTAAGGTTCTTCGCGTAGCTTCGAATTAAACCACATGCTCCACCGCTTGTGCGGGCCCCCGTCAATTCCTTTGA

GTTTCAGCCTTGCGGCCGTACTCCCCAGGCGGAATGCTTAATGCGTTAGCTGCAGCACTGAAGGGCGGAAACCCTCCAAC

ACTTAGCATTCATCGTTTACGGTATGGACTACCAGGGTATCTAATCCTGTTTGCTACCCATACTTTCGAGCCTCAGCGTC

AGTTACAGACCAGACAGCCGCCTTCGCCACTGGTGTTCTTCCATATATCTACGCATTTCACCGCTACACATGGAGTTCCA

CTGTCCTCTTCTGCACTCAAGTTTCCCAGTTTCCGATGCACTTCTTCGGTTGAGCCGAAGGCTTTCACATCAGACTTAAA

AAACCGCCTGCGCTCGCTTTACGCCCAATAAATCCGGACAACGCTTGCCACCTACGTATTACCGCGGCTGCTGGCACGTA

GTTAGCCGTGGCTTTCTGGTTAAATACCGTCAATACCTGAACAGTTACTCTCAGATATGTTCTTCTTTAACAACAGAGTT

TTACGAGCCGAAACCCTTCTTCACTCACGCGGCGTTGCTCCATCAGACTTTCGTCCATTGTGGAAGATTCCCTACTGCTG

CCTCCCGTAGGAGTTTGGGCCGTGTCTCAGTCCCAATGTGGCCGATTACCCTCTCAGGTCGGCTACGTATCATTGCCATG

GTGAGCCGTTACCCCACCATCTAGCTAATACGCCGCGGGACCATCCAAAAGTGATAGCCGAAGCCATCTTTCAAGCTCGG

ACCATGCGGTCCAAGTTGTTATGCGGTATTAGCATCTGTTTCCAGGTGTTATCCCCCGCTTCTGGGCAGGTTTCCCACGT

GTTACTCACCAGTTCGCCACTCACTCAAATGTAAATCATGATGCAAGCACCAATCAATACCAGAGTTCGTTCGACTTGCA

TGTATTAGGCACGCCGCCAGCGTTCGTCCTGAGCCAGGATCAAACTCTCAAATTAA

>CP016071.1 Lactobacillus plantarum strain NCU116, complete genome

TTCCTTAGAAAGGAGGTGATCCAGCCGCAGGTTCTCCTACGGCTACCTTGTTACGACTTCACCCTAATCATCTGTCCCAC

CTTAGGCGGCTGGTTCCTAAAAGGTTACCCCACCGACTTTGGGTGTTACAAACTCTCATGGTGTGACGGGCGGTGTGTAC

AAGGCCCGGGAACGTATTCACCGCGGCATGCTGATCCGCGATTACTAGCGATTCCGACTTCATGTAGGCGAGTTGCAGCC

TACAATCCGAACTGAGAATGGCTTTAAGAGATTAGCTTACTCTCGCGAGTTCGCAACTCGTTGTACCATCCATTGTAGCA

CGTGTGTAGCCCAGGTCATAAGGGGCATGATGATTTGACGTCATCCCCACCTTCCTCCGGTTTGTCACCGGCAGTCTCAC

CAGAGTGCCCAACTTAATGCTGGCAACTGATAATAAGGGTTGCGCTCGTTGCGGGACTTAACCCAACATCTCACGACACG

AGCTGACGACAACCATGCACCACCTGTATCCATGTCCCCGAAGGGAACGTCTAATCTCTTAGATTTGCATAGTATGTCAA

GACCTGGTAAGGTTCTTCGCGTAGCTTCGAATTAAACCACATGCTCCACCGCTTGTGCGGGCCCCCGTCAATTCCTTTGA

GTTTCAGCCTTGCGGCCGTACTCCCCAGGCGGAATGCTTAATGCGTTAGCTGCAGCACTGAAGGGCGGAAACCCTCCAAC

ACTTAGCATTCATCGTTTACGGTATGGACTACCAGGGTATCTAATCCTGTTTGCTACCCATACTTTCGAGCCTCAGCGTC

AGTTACAGACCAGACAGCCGCCTTCGCCACTGGTGTTCTTCCATATATCTACGCATTTCACCGCTACACATGGAGTTCCA

CTGTCCTCTTCTGCACTCAAGTTTCCCAGTTTCCGATGCACTTCTTCGGTTAAGCCGAAGGCTTTCACATCAGACTTAAA

AAACCGCCTGCGCTCGCTTTACGCCCAATAAATCCGGACAACGCTTGCCACCTACGTATTACCGCGGCTGCTGGCACGTA

GTTAGCCGTGGCTTTCTGGTTAAATACCGTCAATACCTGAACAGTTACTCTCAGATATGTTCTTCTTTAACAACAGAGTT

TTACGAGCCGAAACCCTTCTTCACTCACGCGGCGTTGCTCCATCAGACTTTCGTCCATTGTGGAAGATTCCCTACTGCTG

CCTCCCGTAGGAGTTTGGGCCGTGTCTCAGTCCCAATGTGGCCGATTACCCTCTCAGGTCGGCTACGTATCATTGCCATG

GTGAGCCGTTACCCCACCATCTAGCTAATACACCGCGGGACCATCCAAAAGTGATAGCCGAAGCCATCTTTCAAGCTCGG

ACCATGCGGTCCAAGTTGTTATGCGGTATTAGCATCTGTTTCCAGGTGTTATCCCCCGCTTCTGGGCAGGTTTCCCACGT

GTTACTCACCAGTTCGCCACTCACTCAAATGTAAATCATGATGCAAGCACCAATCAATACCAGAGTTCGTTCGACTTGCA

TGTATTAGGCACGCCGCCAGCGTTCGTCCTGAGCCAGGATCAAACTCTCAAATTAA

>HE600693.1 Lactobacillus paraplantarum partial 16S rRNA gene, strain BGCG11

CCGCGGTGGTGCTTGCATCATGAATTACATTTGAGTGAGTGGCGAACTGGTGAGTAACACGTGGGAAACCTGCCCAGAAG

CGGGGGATAACACCTGGAAACAGATGCTAATACCGCAAACAACTTGGACCGCAGGTCCGAGTTTGAAAGATGGCTTCGGC

TATCACTTTTGGATGGTCCCGCGGCGTATTAGCTAGATGGTGAGGTAACGGCTCACCATGGCAATGATACGTAGCCGACC

TGAGAGGGTAATCGGCCACATTGGGACTGAGACACGGCCCAAACTCCTACGGGAGGCAGCAGTAGGGAATCTTCCACAAT

GGACGAAAGTCTGATGGAGCAACGCCGCGTGAGTGAAGAAGGGTTTCGGCTCGTAAAACTCTGTTGTTAAAGAAGAACAT

ATCTGAGAGTAACTGTTCAGGTATTGACGGTATTTAACCAGAAAGCCACGGCTAACTACGTGCCAGCAGCCGCGGTAATA

CGTAGGTGGCAAGCGTTGTCCGGATTTATTGGGCGTAAAGCGAGCGCAGGCGGTTTTTTAAGTCTGATGTGAAAGCCTTC

GGCTCAACCGAAGAAGTGCATCGGAAACTGGGAAACTTGAGTGCAGAAGAGGACAGTGGAACTCCATGTGTAGCGGTGAA

ATGCGTAGATATATGGAAGAACACCAGTGGCGAAGGCGGCTGTCTGGTCTGTAACTGACGCTGAGGCTCGAAAGTATGGG

TAGCAAACAGGATTAGATACCCTGGTAGTCCATACCGTAAACGATGAATGCTAAGTGTTGGAGGGTTTCCGCCCTTCAGT

GCTGCAGCTAACGCATTAAGCATTCCGCCTGGGGAGTACGGCCGCAAGGCTGAAACTCAAAGGAATTGACGGGGGCCCGC

ACAAGCGGTGGAGCATGTGGTTTAATTCGAAGCTACGCGAAGAACCTTACCAGGTCTTGACATACTATGCAAATCTAAGA

GATTAGACGTTCCCTTCGGGGACATGGATACAGGTGGTGCATGGTTGTCGTCAGCTCGTGTCGTGAGATGTTGGGTTAAG

TCCCGCAACGAGCGCAACCCTTATTATCAGTTGCCAGCATTAAGTTGGGCACTCTGGTGAGACTGCCGGTGACAAACCGG

AGGAAGGTGGGGATGACGTCAAATCATCATGCCCCTTATGACCTGGGCTACACACGTGCTACAATGGATGGTACAACGAG

TTGCGAACTCGCGAGAGTAAGCTAATCTCTTAAAGCCATTCTCAGTTCGGATTGTAGGCTGCAACTCGCCTACATGAAGT

CGGAATCGCTAGTAATCGCGGATCAGCATGCCGCGGTGAATACGTTCCCGGGCCTTGTACACACCGCCCGTCACACCATG

AGAGTTTGTAACACCCAAAGTCGGTGGGGTAACCTTTTAGGAACCAGCCGTA

>KT324627.1 Lactobacillus paraplantarum strain MRS8 16S ribosomal RNA gene, partial sequence

TGGTGCTTGCATCATGAATTACATTTGAGTGAGTGGCGAACTGGTGAGTAACACGTGGGAAACCTGCCCAGAAGCGGGGG

ATAACACCTGGAAACAGATGCTAATACCGCATAACAACTTGGACCGCATGGTCCGAGTTTGAAAGATGGCTTCGGCTATC

ACTTTTGGATGGTCCCGCGGCGTATTAGCTAGATGGTGAGGTAACGGCTCACCATGGCAATGATACGTAGCCGACCTGAG

AGGGTAATCGGCCACATTGGGACTGAGACACGGCCCAAACTCCTACGGGAGGCAGCAGTAGGGAATCTTCCACAATGGAC

GAAAGTCTGATGGAGCAACGCCGCGTGAGTGAAGAAGGGTTTCGGCTCGTAAAACTCTGTTGTTAAAGAAGAACATATCT

GAGAGTAACTGTTCAGGTATTGACGGTATTTAACCAGAAAGCCACGGCTAACTACGTGCCAGCAGCCGCGGTAATACGTA

GGTGGCAAGCGTTGTCCGGATTTATTGGGCGTAAAGCGAGCGCAGGCGGTTTTTTAAGTCTGATGTGAAAGCCTTCGGCT

CAACCGAAGAAGTGCATCGGAAACTGGGAAACTTGAGTGCAGAAGAGGACAGTGGAACTCCATGTGTAGCGGTGAAATGC

GTAGATATATGGAAGAACACCAGTGGCGAAGGCGGCTGTCTGGTCTGTAACTGACGCTGAGGCTCGAAAGTATGGGTAGC

AAACAGGATTAGATACCCTGGTAGTCCATACCGTAAACGATGAATGCTAAGTGTTGGAGGGTTTCCGCCCTTCAGTGCTG

CAGCTAACGCATTAAGCATTCCGCCTGGGGAGTACGGCCGCAAGGCTGAAACTCAAAGGAATTGACGGGGGCCCGCACAA

GCGGTGGAGCATGTGGTTTAATTCGAAGCTACGCGAAGAACCTTACCAGGTCTTGACATACTATGCAAATCTAAGAGATT

AGACGTTCCCTTCGGGGACATGGATACAGGTGGTGCATGGTTGTCGTCAGCTCGTGTCGTGAGATGTTGGGTTAAGTCCC

GCAACGAGCGCAACCCTTATTATCAGTTGCCAGCATTAAGTTGGGCACTCTGGTGAGACTGCCGGTGACAAACCGGAGGA

AGGTGGGGATGACGTCAAATCATCATGCCCCTTATGACCTGGGCTACACACGTGCTACAATGGATGGTACAACGAGTTGC

GAACTCGCGAGAGTAAGCTAATCTCTTAAAGCCATTCTCAGTTCGGATTGTAGGCTGCAACTCGCCTACATGAAGTCGGA

ATCGCTAGTAATCGCGGATCAGCATGCCGCGGTGAATACGTTCCCGGGCCTTGTACACACCGCCCGTCACACCATGAGAG

TTTGTAACACCCAAAGTCGGTGGGGTAACCTTTTAGGAACCAGCCG

>KF569800.1 Lactobacillus paraplantarum strain FT259 16S ribosomal RNA gene, partial sequence

AGTCGTACGAACTCTGTGTAATGATTGGTGCTTGCATCATGAGTTACATTTGAGTGAGTGGCGAACTGGTGAGTAACACG

TGGGAAACCTGCCCAGAAGCGGGGGATAACACSTGGAAACAGATGCTAATACCGCRTAACAACTTGGACCGCATGGTCCG

AGTTTGAAAGATGGCTTCGGCTATCACTTKTGGATGGTCCCGCGGCGTATTAGCTAGATGGTGAGGTAACGGCTCACCAT

GGSAATGATACGTAGCCGACCTGAGAGGGTAATCGGCCACATTGGGACTGAGACACGGCCCAAACTCCTACGGGAGGCAG

CAGTAGGGAATCTTCCACAATGGACGAAAGTCTGATGGAGCAACGCCGCGTGAGTGAAGAAGGGTTTCGGCTCGTAAAAC

TCTGTTGTTAAAGAAGAACATATCTGAGAGTAACTGTTCAGGTATTGACGGTATTTAACCAGAAAGCCACGGCTAACTAC

GTGCCAGCAGCCGCGGTAATACGTAGGTGGCAAGCGTTGTCCGGATTTATTGGGCGTAAAGCGAGCGCAGGCGGTTTTTT

AAGTCTGATGTGAAAGCCTTCGGCTCAACCGAAGAAGTGCATCGGAAACTGGGAAACTTGAGTGCAGAAGAGGACAGTGG

AACTCCATGTGTAGCGGTGAAATGCGTAGATATATGGAAGAACACCAGTGGCGAAGGCGGCTGTCTGGTCTGTAACTGAC

GCTGAGGCTCGAAAGTATGGGTAGCAAACAGGATTAGATACCTGGTAGTCCATACCGTAAACGATGAATGCTAAGTGTTG

GAGGGTTTCCGCCCTTCAGTGCTGCAGCTAACGCATTAAGCATTCCGCCTGGGGAGTACGGCCGCAAGGCTGAAACTCAA

AGGAATTGACGGGGGCCCGCACAAGCGGTGGAGCATGTGGTTTAATTCGAAGCTACGCGAAGAACCTTACCAGGTCTTGA

CATACTATGCAAATCTAAGAGATTAGACGTTCCCTTCGGGGACATGGATACAGGTGGTGCATGGTTGTCGTCAGCTCGTG

TCGTGAGATGTTGGGTTAAGTCCCGCAACGAGCGCAACCCTTATTATCAGTTGCCAGCATTAAGTTGGGCACTCTGGTGA

GACTGCCGGTGACAAACCGGAGGAAGGTGGGGATGACGTCAAATCATCATGCCCCTTATGACCTGGGCTACACACGTGCT

ACAATGGATGGTACAACGAGTTGCGAACTCGCGAGAGTAAGCTAATCTCTTAAAGCCATTCTCAGTTCGGATTGTAGGCT

GCAACTCGCCTACATGAAGTCGGAATCGCTAGTAATCGCGGATCAGCATGCCGCGGTGAATACGTTCCCGGGCCTTGTAC

ACACCGCCCGTCACACCATGAGAGTTTGTAACACCCAAAGTCGGTGGGGTAACC

>KT324627.1 Lactobacillus paraplantarum strain MRS8 16S ribosomal RNA gene, partial sequence

AGTCGAACGAACTCTGGTAATGATTGGTGCTTGCATCATGAATTACATTTGAGTGAGTGGCGAACTGGTGAGTAACACGT

GGGAAACCTGCCCAGAAGCGGGGGATAACACCTGGAAACAGATGCTAATACCGCATAACAACTTGGACCGCATGGTCCGA

GTTTGAAAGATGGCTTCGGCTATCACTTTTGGATGGTCCCGCGGCGTATTAGCTAGATGGTGAGGTAACGGCTCACCATG

GCAATGATACGTAGCCGACCTGAGAGGGTAATCGGCCACATTGGGACTGAGACACGGCCCAAACTCCTACGGGAGGCAGC

AGTAGGGAATCTTCCACAATGGACGAAAGTCTGATGGAGCAACGCCGCGTGAGTGAAGAAGGGTTTCGGCTCGTAAAACT

CTGTTGTTAAAGAAGAACATATCTGAGAGTAACTGTTCAGGTATTGACGGTATTTAACCAGAAAGCCACGGCTAACTACG

TGCCAGCAGCCGCGGTAATACGTAGGTGGCAAGCGTTGTCCGGATTTATTGGGCGTAAAGCGAGCGCAGGCGGTTTTTTA

AGTCTGATGTGAAAGCCTTCGGCTCAACCGAAGAAGTGCATCGGAAACTGGGAAACTTGAGTGCAGAAGAGGACAGTGGA

ACTCCATGTGTAGCGGTGAAATGCGTAGATATATGGAAGAACACCAGTGGCGAAGGCGGCTGTCTGGTCTGTAACTGACG

CTGAGGCTCGAAAGTATGGGTAGCAAACAGGATTAGATACCCTGGTAGTCCATACCGTAAACGATGAATGCTAAGTGTTG

GAGGGTTTCCGCCCTTCAGTGCTGCAGCTAACGCATTAAGCATTCCGCCTGGGGAGTACGGCCGCAAGGCTGAAACTCAA

AGGAATTGACGGGGGCCCGCACAAGCGGTGGAGCATGTGGTTTAATTCGAAGCTACGCGAAGAACCTTACCAGGTCTTGA

CATACTATGCAAATCTAAGAGATTAGACGTTCCCTTCGGGGACATGGATACAGGTGGTGCATGGTTGTCGTCAGCTCGTG

TCGTGAGATGTTGGGTTAAGTCCCGCAACGAGCGCAACCCTTATTATCAGTTGCCAGCATTAAGTTGGGCACTCTGGTGA

GACTGCCGGTGACAAACCGGAGGAAGGTGGGGATGACGTCAAATCATCATGCCCCTTATGACCTGGGCTACACACGTGCT

ACAATGGATGGTACAACGAGTTGCGAACTCGCGAGAGTAAGCTAATCTCTTAAAGCCATTCTCAGTTCGGATTGTAGGCT

GCAACTCGCCTACATGAAGTCGGAATCGCTAGTAATCGCGGATCAGCATGCCGCGGTGAATACGTTCCCGGGCCTTGTAC

ACACCGCCCGTCACACCATGAGAGTTTGTAACACCCAAAGTCGGTGGGGTAACC

>KC422317.1 Lactobacillus pentosus strain KCC-2 16S ribosomal RNA gene, complete sequence

GGGTGCTATAATGCAAGTCGACGAACTCTGGTATTGATTGGTGCTTGCATCATGATTTACATTTGAGTGAGTGGCGAACT

GGTGAGTAACACGTGGGAAACCTGCCCAGAAGCGGGGGATAACACCTGGAAACAGATGCTAATACCGCATAACAACTTGG

ACCGCATGGTCCGAGCTTGAAAGATGGCTTCGGCTATCACTTTTGGATGGTCCCGCGGCGTATTAGCTAGATGGTGGGGT

AACGGCTCACCATGGCAATGATACGTAGCCGACCTGAGAGGGTAATCGGCCACATTGGGACTGAGACACGGCCCAAACTC

CTACGGGAGGCAGCAGTAGGGAATCTTCCACAATGGACGAAAGTCTGATGGAGCAACGCCGCGTGAGTGAAGAAGGGTTT

CGGCTCGTAAAACTCTGTTGTTAAAGAAGAACATATCTGAGAGTAACTGTTCAGGTATTGACGGTATTTAACCAGAAAGC

CACGGCTAACTACGTGCCAGCAGCCGCGGTAATACGTAGGTGGCAAGCGTTGTCCGGATTTATTGGGCGTAAAGCGAGCG

CAGGCGGTTTTTTAAGTCTGATGTGAAAGCCTTCGGCTCAACCGAAGAAGTGCATCGGAAACTGGGAAACTTGAGTGCAG

AAGAGGACAGTGGAACTCCATGTGTAGCGGTGAAATGCGTAGATATATGGAAGAACACCAGTGGCGAAGGCGGCTGTCTG

GTCTGTAACTGACGCTGAGGCTCGAAAGTATGGGTAGCAAACAGGATTAGATACCCTGGTAGTCCATACCGTAAACGATG

AATGCTAAGTGTTGGAGGGTTTCCGCCCTTCAGTGCTGCAGCTAACGCATTAAGCATTCCGCCTGGGGAGTACGGCCGCA

AGGCTGAAACTCAAAGGAATTGACGGGGGCCCGCACAAGCGGTGGAGCATGTGGTTTAATTCGAAGCTACGCGAAGAACC

TTACCAGGTCTTGACATACTATGCAAATCTAAGAGATTAGACGTTCCCTTCGGGGACATGGATACAGGGGGTGCATGGTT

GTCGTCAGCTCGTGTCGTGAGATGTTGGGTTAAGTCCCGCAACGAGCGCAACCCTTATTATCAGTTGCCAGCATTAAGTT

GGGCACTCTGGTGAGACTGCCGGTGACAAACCGGAGGAAGGTGGGGATGACGTCAAATCATCATGCCCCTTATGACCTGG

GCTACACACGTGCTACAATGGATGGTACAACGAGTTGCGAACTCGCGAGAGTAAGCTAATCTCTTAAAGCCATTCTCAGT

TCGGATTGTAGGCTGCAACTCGCCTACATGAAGTCGGAATCGCTAGTAATCGCGGATCAGCATGCCGCGGTGAATACGTT

CCCGGGCCTTGTACACACCGCCCGTCACACCATGAGAGTTTGTAACACCCAAAGTCGGTGGGGTAACCTTTTAGGAACCA

GCCGCCTAATGGTGACA

>AB488810.1 Lactobacillus pentosus gene for 16S ribosomal RNA, partial sequence, strain: YM2-2

CTAATACATGCAAGTCGAACGAACTCTGGTATTGATTGGTGCTTGCATCATGATTTACATTTGAGTGAGTGGCGAACTGG

TGAGTAACACGTGGGAAACCTGCCCAGAAGCGGGGGATAACACCTGGAAACAGATGCTAATACCGCATAACAACTTGGAC

CGCATGGTCCGAGTTTGAAAGATGGCTTCGGCTATCACTTTTGGATGGTCCCGCGGCGTATTAGCTAGATGGTGGGGTAA

CGGCTCACCATGGCAATGATACGTAGCCGACCTGAGAGGGTAATCGGCCACATTGGGACTGAGACACGGCCCAAACTCCT

ACGGGAGGCAGCAGTAGGGAATCTTCCACAATGGACGAAAGTCTGATGGAGCAACGCCGCGTGAGTGAAGAAGGGTTTCG

GCTCGTAAAACTCTGTTGTTAAAGAAGAACATATCTGAGAGTAACTGTTCAGGTATTGACGGTATTTAACCAGAAAGCCA

CGGCTAACTACGTGCCAGCAGCCGCGGTAATACGTAGGTGGCAAGCGTTGTCCGGATTTATTGGGCGTAAAGCGAGCGCA

GGCGGTTTTTTAAGTCTGATGTGAAAGCCTTCGGCTCAACCGAAGAAGTGCATCGGAAACTGGGAAACTTGAGTGCAGAA

GAGGACAGTGGAACTCCATGTGTAGCGGTGAAATGCGTAGATATATGGAAGAACACCAGTGGCGAAGGCGGCTGTCTGGT

CTGTAACTGACGCTGAGGCTCGAAAGTATGGGTAGCAAACAGGATTAGATACCCTGGTAGTCCATACCGTAAACGATGAA

TGCTAAGTGTTGGAGGGTTTCCGCCCTTCAGTGCTGCAGCTAACGCATTAAGCATTCCGCCTGGGGAGTACGGCCGCAAG

GCTGAAACTCAAAGGAATTGACGGGGGCCCGCACAAGCGGTGGAGCATGTGGTTTAATTCGAAGCTACGCGAAGAACCTT

ACCAGGTCTTGACATACTATGCAAATCTAAGAGATTAGACGTTCCCTTCGGGGACATGGATACAGGTGGTGCATGGTTGT

CGTCAGCTCGTGTCGTGAGATGTTGGGTTAAGTCCCGCAACGAGCGCAACCCTTATTATCAGTTGCCAGCATTAAGTTGG

GCACTCTGGTGAGACTGCCGGTGACAAACCGGAGGAAGGTGGGGATGACGTCAAATCATCATGCCCCTTATGACCTGGGC

TACACACGTGCTACAATGGATGGTACAACGAGTTGCGAACTCGCGAGAGTAAGCTAATCTCTTAAAGCCATTCTCAGTTC

GGATTGTAGGCTGCAACTCGCCTACATGAAGTCGGAATCGCTAGTAATCGCGGATCAGCATGCCGCGGTGAATACGTTCC

CGGGCCTTGTACACACCGCCCGTCACACCATGAGAGTTTGTAACACCCAAAGTCGGTGGGGTAACCTTTTAGGAACCAGC

CGCCTAAGGTGGGACAGATGATTAGGGTGAAGTCGTAACAAGGTAGCCGTAGGAGAACCTGCGGTTG

>CP021997.1 Lactobacillus plantarum strain LPL-1, complete genome

CAGCCGCAGGTTCTCCTACGGCTACCTTGTTACGACTTCACCCTAATCATCTGTCCCACCTTAGGCGGCTGGTTCCTAAA

AGGTTACCCCACCGACTTTGGGTGTTACAAACTCTCATGGTGTGACGGGCGGTGTGTACAAGGCCCGGGAACGTATTCAC

CGCGGCATGCTGATCCGCGATTACTAGCGATTCCGACTTCATGTAGGCGAGTTGCAGCCTACAATCCGAACTGAGAATGG

CTTTAAGAGATTAGCTTACTCTCGCGAGTTCGCAACTCGTTGTACCATCCATTGTAGCACGTGTGTAGCCCAGGTCATAA

GGGGCATGATGATTTGACGTCATCCCCACCTTCCTCCGGTTTGTCACCGGCAGTCTCACCAGAGTGCCCAACTTAATGCT

GGCAACTGATAATAAGGGTTGCGCTCGTTGCGGGACTTAACCCAACATCTCACGACACGAGCTGACGACAACCATGCACC

ACCTGTATCCATGTCCCCGAAGGGAACGTCTAATCTCTTAGATTTGCATAGTATGTCAAGACCTGGTAAGGTTCTTCGCG

TAGCTTCGAATTAAACCACATGCTCCACCGCTTGTGCGGGCCCCCGTCAATTCCTTTGAGTTTCAGCCTTGCGGCCGTAC

TCCCCAGGCGGAATGCTTAATGCGTTAGCTGCAGCACTGAAGGGCGGAAACCCTCCAACACTTAGCATTCATCGTTTACG

GTATGGACTACCAGGGTATCTAATCCTGTTTGCTACCCATACTTTCGAGCCTCAGCGTCAGTTACAGACCAGACAGCCGC

CTTCGCCACTGGTGTTCTTCCATATATCTACGCATTTCACCGCTACACATGGAGTTCCACTGTCCTCTTCTGCACTCAAG

TTTCCCAGTTTCCGATGCACTTCTTCGGTTGAGCCGAAGGCTTTCACATCAGACTTAAAAAACCGCCTGCGCTCGCTTTA

CGCCCAATAAATCCGGACAACGCTTGCCACCTACGTATTACCGCGGCTGCTGGCACGTAGTTAGCCGTGGCTTTCTGGTT

AAATACCGTCAATACCTGAACAGTTACTCTCAGATATGTTCTTCTTTAACAACAGAGTTTTACGAGCCGAAACCCTTCTT

CACTCACGCGGCGTTGCTCCATCAGACTTTCGTCCATTGTGGAAGATTCCCTACTGCTGCCTCCCGTAGGAGTTTGGGCC

GTGTCTCAGTCCCAATGTGGCCGATTACCCTCTCAGGTCGGCTACGTATCATTGCCATGGTGAGCCGTTACCCCACCATC

TAGCTAATACGCCGCGGGACCATCCAAAAGTGATAGCCGAAGCCATCTTTCAAGCTCGGACCATGCGGTCCAAGTTGTTA

TGCGGTATTAGCATCTGTTTCCAGGTGTTATCCCCCGCTTCTGGGCAGGTTTCCCACGTGTTACTCACCAGTTCGCCACT

CACTCAAATGTAAATCATGATGCAAGCACCAATCAATACCAGAGTTCGTTCGACTTGCATGTATTAG

>CP021997.1 Lactobacillus plantarum strain LPL-1, complete genome

CAGCCGCAGGTTCTCCTACGGCTACCTTGTTACGACTTCACCCTAATCATCTGTCCCACCTTAGGCGGCTGGTTCCTAAA

AGGTTACCCCACCGACTTTGGGTGTTACAAACTCTCATGGTGTGACGGGCGGTGTGTACAAGGCCCGGGAACGTATTCAC

CGCGGCATGCTGATCCGCGATTACTAGCGATTCCGACTTCATGTAGGCGAGTTGCAGCCTACAATCCGAACTGAGAATGG

CTTTAAGAGATTAGCTTACTCTCGCGAGTTCGCAACTCGTTGTACCATCCATTGTAGCACGTGTGTAGCCCAGGTCATAA

GGGGCATGATGATTTGACGTCATCCCCACCTTCCTCCGGTTTGTCACCGGCAGTCTCACCAGAGTGCCCAACTTAATGCT

GGCAACTGATAATAAGGGTTGCGCTCGTTGCGGGACTTAACCCAACATCTCACGACACGAGCTGACGACAACCATGCACC

ACCTGTATCCATGTCCCCGAAGGGAACGTCTAATCTCTTAGATTTGCATAGTATGTCAAGACCTGGTAAGGTTCTTCGCG

TAGCTTCGAATTAAACCACATGCTCCACCGCTTGTGCGGGCCCCCGTCAATTCCTTTGAGTTTCAGCCTTGCGGCCGTAC

TCCCCAGGCGGAATGCTTAATGCGTTAGCTGCAGCACTGAAGGGCGGAAACCCTCCAACACTTAGCATTCATCGTTTACG

GTATGGACTACCAGGGTATCTAATCCTGTTTGCTACCCATACTTTCGAGCCTCAGCGTCAGTTACAGACCAGACAGCCGC

CTTCGCCACTGGTGTTCTTCCATATATCTACGCATTTCACCGCTACACATGGAGTTCCACTGTCCTCTTCTGCACTCAAG

TTTCCCAGTTTCCGATGCACTTCTTCGGTTGAGCCGAAGGCTTTCACATCAGACTTAAAAAACCGCCTGCGCTCGCTTTA

CGCCCAATAAATCCGGACAACGCTTGCCACCTACGTATTACCGCGGCTGCTGGCACGTAGTTAGCCGTGGCTTTCTGGTT

AAATACCGTCAATACCTGAACAGTTACTCTCAGATATGTTCTTCTTTAACAACAGAGTTTTACGAGCCGAAACCCTTCTT

CACTCACGCGGCGTTGCTCCATCAGACTTTCGTCCATTGTGGAAGATTCCCTACTGCTGCCTCCCGTAGGAGTTTGGGCC

GTGTCTCAGTCCCAATGTGGCCGATTACCCTCTCAGGTCGGCTACGTATCATTGCCATGGTGAGCCGTTACCTCACCATC

TAGCTAATACGCCGCGGGACCATCCAAAAGTGATAGCCGAAGCCATCTTTCAAACTCGGACCATGCGGTCCAAGTTGTTA

TGCGGTATTAGCATCTGTTTCCAGGTGTTATCCCCCGCTTCTGGGCAGGTTTCCCACGTGTTACTCACCAGTTCGCCACT

CACTCAAATGTAAATCATGATGCAAGCACCAATCAATACCAGAGTTCGTTCGACTTGCATGTATTAG

>CP021997.1 Lactobacillus plantarum strain LPL-1, complete genome

CTAATACATGCAAGTCGAACGAACTCTGGTATTGATTGGTGCTTGCATCATGATTTACATTTGAGTGAGTGGCGAACTGG

TGAGTAACACGTGGGAAACCTGCCCAGAAGCGGGGGATAACACCTGGAAACAGATGCTAATACCGCATAACAACTTGGAC

CGCATGGTCCGAGTTTGAAAGATGGCTTCGGCTATCACTTTTGGATGGTCCCGCGGCGTATTAGCTAGATGGTGAGGTAA

CGGCTCACCATGGCAATGATACGTAGCCGACCTGAGAGGGTAATCGGCCACATTGGGACTGAGACACGGCCCAAACTCCT

ACGGGAGGCAGCAGTAGGGAATCTTCCACAATGGACGAAAGTCTGATGGAGCAACGCCGCGTGAGTGAAGAAGGGTTTCG

GCTCGTAAAACTCTGTTGTTAAAGAAGAACATATCTGAGAGTAACTGTTCAGGTATTGACGGTATTTAACCAGAAAGCCA

CGGCTAACTACGTGCCAGCAGCCGCGGTAATACGTAGGTGGCAAGCGTTGTCCGGATTTATTGGGCGTAAAGCGAGCGCA

GGCGGTTTTTTAAGTCTGATGTGAAAGCCTTCGGCTCAACCGAAGAAGTGCATCGGAAACTGGGGAACTTGAGTGCAGAA

GAGGACAGTGGAACTCCATGTGTAGCGGTGAAATGCGTAGATATATGGAAGAACACCAGTGGCGAAGGCGGCTGTCTGGT

CTGTAACTGACGCTGAGGCTCGAAAGTATGGGTAGCAAACAGGATTAGATACCCTGGTAGTCCATACCGTAAACGATGAA

TGCTAAGTGTTGGAGGGTTTCCGCCCTTCAGTGCTGCAGCTAACGCATTAAGCATTCCGCCTGGGGAGTACGGCCGCAAG

GCTGAAACTCAAAGGAATTGACGGGGGCCCGCACAAGCGGTGGAGCATGTGGTTTAATTCGAAGCTACGCGAAGAACCTT

ACCAGGTCTTGACATACTATGCAAATCTAAGAGATTAGACGTTCCCTTCGGGGACATGGATACAGGTGGTGCATGGTTGT

CGTCAGCTCGTGTCGTGAGATGTTGGGTTAAGTCCCGCAACGAGCGCAACCCTTATTATCAGTTGCCAGCATTAAGTTGG

GCACTCTGGTGAGACTGCCGGTGACAAACCGGAGGAAGGTGGGGATGACGTCAAATCATCATGCCCCTTATGACCTGGGC

TACACACGTGCTACAATGGATGGTACAACGAGTTGCGAACTCGCGAGAGTAAGCTAATCTCTTAAAGCCATTCTCAGTTC

GGATTGTAGGCTGCAACTCGCCTACATGAAGTCGGAATCGCTAGTAATCGCGGATCAGCATGCCGCGGTGAATACGTTCC

CGGGCCTTGTACACACCGCCCGTCACACCATGAGAGTTTGTAACACCCAAAGTCGGTGGGGTAACCTTTTAGGAACCAGC

CGCCTAAGGTGGGACAGATGATTAGGGTGAAGTCGTAACAAGGTAGCCGTAGGAGAACCTGCGG

>CP021997.1 Lactobacillus plantarum strain LPL-1, complete genome

CTAATACATGCAAGTCGAACGAACTCTGGTATTGATTGGTGCTTGCATCATGATTTACATTTGAGTGAGTGGCGAACTGG

TGAGTAACACGTGGGAAACCTGCCCAGAAGCGGGGGATAACACCTGGAAACAGATGCTAATACCGCATAACAACTTGGAC

CGCATGGTCCGAGTTTGAAAGATGGCTTCGGCTATCACTTTTGGATGGTCCCGCGGCGTATTAGCTAGATGGTGAGGCAA

CGGCTCACCATGGCAATGATACGTAGCCGACCTGAGAGGGTAATCGGCCACATTGGGACTGAGACACGGCCCAAACTCCT

ACGGGAGGCAGCAGTAGGGAATCTTCCACAATGGACGAAAGTCTGATGGAGCAACGCCGCGTGAGTGAAGAAGGGTTTCG

GCTCGTAAAACTCTGTTGTTAAAGAAGAACATATCTGAGAGTAACTGTTCAGGTATTGACGGTATTTAACCAGAAAGCCA

CGGCTAACTACGTGCCAGCAGCCGCGGTAATACGTAGGTGGCAAGCGTTGTCCGGATTTATTGGGCGTAAAGCGAGCGCA

GGCGGTTTTTTAAGTCTGATGTGAAAGCCTTCGGCTCAACCGAAGAAGTGCATCGGAAACTGGGAAACTTGAGTGCAGAA

GAGGACAGTGGAACTCCATGTGTAGCGGTGAAATGCGTAGATATATGGAAGAACACCAGTGGCGAAGGCGGCTGTCTGGT

CTGTAACTGACGCTGAGGCTCGAAAGTATGGGTAGCAAACAGGATTAGATACCCTGGTAGTCCATACCGTAAACGATGAA

TGCTAAGTGTTGGAGGGTTTCCGCCCTTCAGTGCTGCAGCTAACGCATTAAGCATTCCGCCTGGGGAGTACGGCCGCAAG

GCTGAAACTCAAAGGAATTGACGGGGGCCCGCACAAGCGGTGGAGCATGTGGTTTAATTCGAAGCTACGCGAAGAACCTT

ACCAGGTCTTGACATACTATGCAAATCTAAGAGATTAGACGTTCCCTTCGGGGACATGGATACAGGTGGTGCATGGTTGT

CGTCAGCTCGTGTCGTGAGATGTTGGGTTAAGTCCCGCAACGAGCGCAACCCTTATTATCAGTTGCCAGCATTAAGTTGG

GCACTCTGGTGAGACTGCCGGTGACAAACCGGAGGAAGGTGGGGATGACGTCAAATCATCATGCCCCTTATGACCTGGGC

TACACACGTGCTACAATGGATGGTACAACGAGTTGCGAACTCGCGAGAGTAAGCTAATCTCTTAAAGCCATTCTCAGTTC

GGATTGTAGGCTGCAACTCGCCTACATGAAGTCGGAATCGCTAGTAATCGCGGATCAGCATGCCGCGGTGAATACGTTCC

CGGGCCTTGTACACACCGCCCGTCACACCATGAGAGTTTGTAACACCCAAAGTCGGTGGGGTAACCTTTTAGGAACCAGC

CGCCTAAGGTGGGACAGATGATTAGGGTGAAGTCGTAACAAGGTAGCCGTAGGAGAACCTGCGG

>CP021997.1 Lactobacillus plantarum strain LPL-1, complete genome

CTAATACATGCAAGTCGAACGAACTCTGGTATTGATTGGTGCTTGCATCATGATTTGCATTTGAGTGAGTGGCGAACTGG

TGAGTAACACGTGGGAAACCTGCCCAGAAGCGGGGGATAACACCTGGAAACAGATGCTAATACCGCATAACAACTTGGAC

CGCATGGTCCGAGTTTGAAAGATGGCTTCGGCTATCACTTTTGGATGGTCCCGCGGCGTATTAGCTAGATGGTGAGGTAA

CGGCTCACCATGGCAATGATACGTAGCCGACCTGAGAGGGTAATCGGCCACATTGGGACTGAGACACGGCCCAAACTCCT

ACGGGAGGCAGCAGTAGGGAATCTTCCACAATGGACGAAAGTCTGATGGAGCAACGCCGCGTGAGTGAAGAAGGGTTTCG

ACTCGTAAAACTCTGTTGTTAAAGAAGAACATATCTGAGAGTAACTGTTCAGGTATTGACGGTATTTAACCAGAAAGCCA

CGGCTAACTACGTGCCAGCAGCCGCGGTAATACGTAGGTGGCAAGCGTTGTCCGGATTTATTGGGCGTAAAGCGAGCGCA

GGCGGTTTTTTAAGTCTGATGTGAAAGCCTTCGGCTCAACCGAAGAAGTGCATCGGAAACTGGGAAACTTGAGTGCAGAA

GAGGACAGTGGAACTCCATGTGTAGCGGTGAAATGCGTAGATATATGGAAGAACACCAGTGGCGAAGGCGGCTGTCTGGT

CTGTAACTGACGCTGAGGCTCGAAAGTATGGGTAGCAAACAGGATTAGATACCCTGGTAGTCCATACCGTAAACGATGAA

TGCTAAGTGTTGGAGGGTTTCCGCCCTTCAGTGCTGCAGCTAACGCATTAAGCATTCCGCCTGGGGAGTACGGCCGCAAG

GCTGAAACTCAAAGGAATTGACGGGGGCCCGCACAAGCGGTGGAGCATGTGGTTTAATTCGAAGCTACGCGAAGAACCTT

ACCAGGTCTTGACATACTATGCAAATCTAAGAGATTAGACGTTCCCTTCGGGGACATGGATACAGGTGGTGCATGGTTGT

CGTCAGCTCGTGTCGTGAGATGTTGGGTTAAGTCCCGCAACGAGCGCAACCCTTATTATCAGTTGCCAGCATTAAGTTGG

GCACTCTGGTGAGACTGCCGGTGACAAACCGGAGGAAGGTGGGGATGACGTCAAATCATCATGCCCCTTATGACCTGGGC

TACACACGTGCTACAATGGATGGTACAACGAGTTGCGAACTCGCGAGAGTAAGCTAATCTCTTAAAGCCATTCTCAGTTC

GGATTGTAGGCTGCAACTCGCCTACATGAAGTCGGAATCGCTAGTAATCGCGGATCAGCATGCCGCGGTGAATACGTTCC

CGGGCCTTGTACACACCGCCCGTCACACCATGAGAGTTTGTAACACCCAAAGTCGGTGGGGTAACCTTTTAGGAACCAGC

CGCCTAAGGTGGGACAGATGATTAGGGTGAAGTCGTAACAAGGTAGCCGTAGGAGAACCTGCGG

>KR149355.1 Lactobacillus pentosus strain Lp-C45 16S ribosomal RNA gene, partial sequence

GACGAACGCTGGCGGCGTGCCTAATACATGCAAGTCGAACGAACTCTGGTATTGATTGGTGCTTGCATCA

TGATTTACATTTGAGTGAGTGGCGAACTGGTGAGTAACACGTGGGAAACCTGCCCAGAAGCGGGGGATAA

CACCTGGAAACAGATGCTAATACCGCATAACAACTTGGACCGCATGGTCCGAGTTTGAAAGATGGCTTCG

GCTATCACTTTTGGATGGTCCCGCGGCGTATTAGCTAGATGGTGGGGTAACGGCTCACCATGGCAATGAT

ACGTAGCCGACCTGAGAGGGTAATCGGCCACATTGGGACTGAGACACGGCCCAAACTCCTACGGGAGGCA

GCAGTAGGGAATCTTCCACAATGGACGAAAGTCTGATGGAGCAACGCCGCGTGAGTGAAGAAGGGTTTCG

GCTCGTAAAACTCTGTTGTTAAAGAAGAACATATCTGAGAGTAACTGTTCAGGTATTGACGGTATTTAAC

CAGAAAGCCACGGCTAACTACGTGCCAGCAGCCGCGGTAATACGTAGGTGGCAAGCGTTGTCCGGATTTA

TTGGGCGTAAAGCGAGCGCAGGCGGTTTTTTAAGTCTGATGTGAAAGCCTTCGGCTCAACCGAAGAAGTG

CATCGGAAACTGGGAAACTTGAGTGCAGAAGAGGACAGTGGAACTCCATGTGTAGCGGTGAAATGCGTAG

ATATATGGAAGAACACCAGTGGCGAAGGCGGCTGTCTGGTCTGTAACTGACGCTGAGGCTCGAAAGTATG

GGTAGCAAACAGGATTAGATACCCTGGTAGTCCATACCGTAAACGATGAATGCTAAGTGTTGGAGGGTTT

CCGCCCTTCAGTGCTGCAGCTAACGCATTAAGCATTCCGCCTGGGGAGTACGGCCGCAAGGCTGAAACTC

AAAGGAATTGACGGGGGCCCGCACAAGCGGTGGAGCATGTGGTTTAATTCGAAGCTACGCGAAGAACCTT

ACCAGGTCTTGACATACTATGCAAATCTAAGAGATTAGACGTTCCCTTCGGGGACATGGATACAGGTGGT

GCATGGTTGTCGTCAGCTCGTGTCGTGAGATGTTGGGTTAAGTCCCGCAACGAGCGCAACCCTTATTATC

AGTTGCCAGCATTAAGTTGGGCACTCTGGTGAGACTGCCGGTGACAAACCGGAGGAAGGTGGGGATGACG

TCAAATCATCATGCCCCTTATGACCTGGGCTACACACGTGCTACAATGGATGGTACAACGAGTTGCGAAC

TCGCGAGAGTAAGCTAATCTCTTAAAGCCATTCTCAGTTCGGATTGTAGGCTGCAACTCGCCTACATGAA

GTCGGAATCGCTAGTAATCGCGGATCAGCATGCCGCGGTGAATACGTTCCCGGGCCTTGTACACACCGCC

CGTCACACCATGAGAGTTTGTAACACCCAAAGTCGGTGGGGTAACCTTTTAGGAACCAGCCGCCTAAGGT

GGGACAGATGATTAGGGTGAAGTCGTAACAAGGTAGCCGTAGGGAGAAC
